# A Spin Glass Model for the Loss Surfaces of Generative Adversarial Networks

**DOI:** 10.1007/s10955-022-02875-w

**Published:** 2022-01-18

**Authors:** Nicholas P. Baskerville, Jonathan P. Keating, Francesco Mezzadri, Joseph Najnudel

**Affiliations:** 1 School of Mathematics, University of Bristol, Fry Building, Bristol, BS8 1UG UK; 2 Mathematical Institute, University of Oxford, Oxford, OX2 6GG UK

**Keywords:** Random matrix theory, Deep learning, Neural networks, Generative adversarial networks, Spin glasses

## Abstract

We present a novel mathematical model that seeks to capture the key design feature of generative adversarial networks (GANs). Our model consists of two interacting spin glasses, and we conduct an extensive theoretical analysis of the complexity of the model’s critical points using techniques from Random Matrix Theory. The result is insights into the loss surfaces of large GANs that build upon prior insights for simpler networks, but also reveal new structure unique to this setting which explains the greater difficulty of training GANs.

## Introduction

By making various modeling assumptions about standard multi-layer perceptron neural networks, [1] argued heuristically that the training loss surfaces of large networks could be modelled by a spherical multi-spin glass. Using theoretical results of [2], they were able to arrive at quantitative asymptotic characterisations, in particular the existence of a favourable ‘banded structure’ of local-optima of the loss. There are clear and acknowledged deficiencies with their assumptions [3] and recent observations have shown that the Hessians of real-world deep neural networks do not behave like random matrices from the Gaussian Orthogonal Ensemble (GOE) of Random Matrix Theory at the macroscopic scale [4–6], despite this being implied by the spin-glass model of [1]. Moreover, there have been questions raised about whether the mean asymptotic properties of loss surfaces for deep neural networks (or energy surfaces of glassy objects) are even relevant practically for gradient-based optimisation in sub-exponential time [7–9], though interpretation of experiments with deep neural networks remains difficult and the discussion about the true shape of their loss surfaces and the implications thereof is far from settled. Nevertheless, spin-glass models present a tractable example of high-dimensional complex random functions that may well provide insights into aspects of deep learning. Rather than trying to improve or reduce the assumptions of [1], various authors have recently opted to skip the direct derivation from a neural network to a statistical physics model, instead proposing simple models designed to capture aspects of training dynamics and studying those directly. Examples include: the modified spin glass model of [10] with some explicitly added ‘signal’; the simple explicitly non-linear model of [11]; the spiked tensor ‘signal-in-noise’ model of [12]. In a slightly different direction, [13] removed one of the main assumptions from the [1] derivation, and in so doing arrived at a deformed spin-glass model. All of this recent activity sits in the context of earlier work connecting spin-glass objects with simple neural networks [14–16] and, more generally, with image reconstruction and other signal processing problems [17].

One area that has not been much explored in the line of the above-mentioned literature is the study of architectural variants. Modern deep learning contains a very large variety of different design choices in network architecture, such as convolutional networks for image and text data (among others) [18, 19], recurrent networks for sequence data [20] and self-attention transformer networks for natural language [21, 22]. Given the ubiquity of convolutional networks, one might seek to study those, presumably requiring consideration of local correlations in data. One could imagine some study of architectural quirks such as residual connections [23], and batch-norm has been considered to some extent by [24]. In this work, we propose a novel model for *generative adversarial networks* (GANs) [25] as two interacting spherical spin glasses. GANs have been the focus of intense research and development in recent years, with a large number of variants being proposed [26–32] and rapid progress particularly in the field of image generation. From the perspective of optimisation, GANs have much in common with other deep neural networks, being complicated high-dimensional functions optimised using local gradient-based methods such as stochastic gradient descent and variants. On the other hand, the adversarial training objective of GANs, with two deep networks competing, is clearly an important distinguishing feature, and GANs are known to be more challenging to train than single deep networks. Our objective is to capture the essential adversarial aspect of GANs in a tractable model of high-dimensional random complexity which, though being a significant simplification, has established connections to neural networks and high dimensional statistics.

Our model is inspired by [1, 12, 33, 34] with spherical multi-spin glasses being used in place of deep neural networks. We thus provide a complicated, random, high-dimensional model with the essential feature of GANs clearly reflected in its construction. By employing standard Kac-Rice complexity calculations [2, 35, 36] we are able to reduce the loss landscape complexity calculation to a random matrix theoretic calculation. We then employ various Random Matrix Theory techniques as in [13] to obtain rigorous, explicit leading order asymptotic results. Our calculations rely on the supersymmetric method in Random Matrix Theory, in particular the approach to calculating limiting spectral densities follows [37] and the calculation also follows [38, 39] in important ways. The greater complexity of the random matrix spectra encountered present some challenges over previous such calculations, which we overcome with a combination of analytical and numerical approaches. Using our complexity results, we are able to draw qualitative implications about GAN loss surfaces analogous to those of [1] and also investigate the effect of a few key design parameters included in the GAN. We compare the effect of these parameters on our spin glass model and also on the results of experiments training real GANs. Our calculations include some novel details, in particular, we use precise sub-leading terms for a limiting spectral density obtained from supersymmetric methods to prove a required concentration result to justify the use of the Coulomb gas approximation. We note that our complexity results could be also be obtained in principle using the methods developed in [40], however our work was completed several months before this pre-print appeared. Our approach for computing the limiting spectral density may nevertheless be the simplest and would be used as input to the results of [40].

The role that statistical physics models such as spherical multi-spin glasses are to ultimately play in the theory of deep learning is not yet clear, with arguments both for and against their usefulness and applicability. We provide a first attempt to model an important architectural feature of modern deep neural networks within the framework of spin glass models and provide a detailed analysis of properties of the resulting loss (energy) surface. Our analysis reveals potential explanations for observed properties of GANs and demonstrates that it may be possible to inform practical hyperparameter choices using models such as ours. Much of the advancement in practical deep learning has come from innovation in network architecture, so if deep learning theory based on simplified physics models like spin-glasses is to keep pace with practical advances in the field, then it will be necessary to account for architectural details within such models. Our work is a first step in that direction and the mathematical techniques used may prove more widely valuable.

The paper is structured as follows: in Sect. 2 we introduce the interacting spin glass model; in Sect. 3 we use a Kac-Rice formula to derive random matrix expressions for the asymptotic complexity of our model; in Sect. 4 we derive the limiting spectral density of the relevant random matrix ensemble; in Sect. 5 we use the Coulomb gas approximation to compute the asymptotic complexity, and legitimise its use by proving a concentration result; in Sect. 6 we derive some implications of our model for GAN training and compare to experimental results from real GANs; in Sect. 7 we conclude. All code used for numerical calculations of our model, training real GANs, analysing the results and generating plots is made available^1^.

## An Interacting Spin Glass Model

We use multi-spin glasses in high dimensions as a toy model for neural network loss surfaces without any further justification, beyond that found in [1, 13]. GANs are composed of two networks: *generator* (*G*) and *discriminator* (*D*). *G* is a map $\mathbb {R}^m\rightarrow \mathbb {R}^d$ and *D* is a map $\mathbb {R}^d\rightarrow \mathbb {R}$. *G*’s purpose is to generate synthetic data samples by transforming random input noise, while *D*’s is to distinguish between real data samples and those generated by *G*. Given some probability distribution $\mathbb {P}_{data}$ on some $\mathbb {R}^d$, GANs have the following minimax training objective

1 $$\begin{aligned} \min _{\varTheta _G}\max _{\varTheta _D}\left\{ \mathbb {E}_{{\varvec{x}}\sim \mathbb {P}_{data}} \log D\left( {\varvec{x}}\right) + \mathbb {E}_{{\varvec{z}}\sim \mathcal {N}\left( 0, \sigma _z^2\right) }\log \left( 1 - D(G({\varvec{z}}))\right) \right\} , \end{aligned}$$

where $\varTheta _D, \varTheta _G$ are the parameters of the discriminator and generator respectively. With ${\varvec{z}}\sim \mathcal {N}(0, \sigma _z^2)$, $G({\varvec{z}})$ has some probability distribution $\mathbb {P}_{gen}$. When successfully trained, the initially unstructured $\mathbb {P}_{gen}$ examples are easily distinguished by *D*, this in turn drives improvements in *G*, bring $\mathbb {P}_{gen}$ closer to $\mathbb {P}_{data}$. Ultimately, the process successfully terminates when $\mathbb {P}_{gen}$ is very close to $\mathbb {P}_{data}$ and *D* performs little better than random at the distinguishing task. To construct our model, we introduce two spin glasses:

2 $$\begin{aligned} \ell ^{(D)}({\varvec{w}}^{(D)})&= \sum _{i_1,\ldots , i_p=1}^{N_D} X_{i_1,\ldots , i_p} \prod _{k=1}^p w^{(D)}_{i_k} \end{aligned}$$

3 $$\begin{aligned} \ell ^{(G)}({\varvec{w}}^{(D)}, {\varvec{w}}^{(G)})&= \sum _{i_1,\ldots , i_{p+q}=1}^{N_D+N_G} Z_{i_1,\ldots , i_{p+q}} \prod _{k=1}^{p+q} w_k \end{aligned}$$

where ${\varvec{w}}^T = ({{\varvec{w}}^{(D)}}^T, {{\varvec{w}}^{(G)}}^T)$, all the $X_{i_1,\ldots , i_p}$ are i.i.d. $\mathcal {N}(0,1)$ and $Z_{j_1,\ldots , j_{p+q}}$ are similarly i.i.d. $\mathcal {N}(0,1)$. We then define the models for the discriminator and generator losses:

4 $$\begin{aligned} L^{(D)}({\varvec{w}}^{(D)}, {\varvec{w}}^{(G)})&= \ell ^{(D)}({\varvec{w}}^{(D)}) - \sigma _z\ell ^{(G)}({\varvec{w}}^{(D)}, {\varvec{w}}^{(G)}), \end{aligned}$$

5 $$\begin{aligned} L^{(G)}({\varvec{w}}^{(D)}, {\varvec{w}}^{(G)})&= \sigma _z \ell ^{(G)}({\varvec{w}}^{(D)},{\varvec{w}}^{(G)}). \end{aligned}$$

$\ell ^{(D)}$ plays the role of the loss of the discriminator network when trying to classify genuine examples as such. $\ell ^{(G)}$ plays the role of loss of the discriminator when applied to samples produced by the generator, hence the sign difference between $L^{(D)}$ and $L^{(G)}$. ${\varvec{w}}^{(D)}$ are the weights of the discriminator, and ${\varvec{w}}^{(G)}$ the weights of the generator. The $X_{{\varvec{i}}}$ are surrogates for the training data (i.e. samples from $\mathbb {P}_{data}$) and the $Z_{{\varvec{j}}}$ are surrogates for the noise distribution of the generator. For convenience, we have chosen to pull the $\sigma _z$ scale outside of the $Z_{{\varvec{j}}}$ and include it as a constant multiplier in (4)–(5). In reality, we should like to keep $Z_{{\varvec{j}}}$ as i.i.d. $\mathcal {N}(0,1)$ but take $X_{{\varvec{i}}}$ to have some other more interesting distribution, e.g. normally or uniformly distributed on some manifold. Using [*x*] to denote the integer part of *x*, we take $N_D = [\kappa N], N_G = [\kappa ' N]$ for fixed $\kappa \in (0,1)$, $\kappa '=1-\kappa$, and study the regime $N\rightarrow \infty$. Note that there is no need to distinguish between $[\kappa N]$ and $\kappa N$ in the $N\rightarrow \infty$ limit.

### Remark 1

Our model is not supposed to have any direct relationship to GANs. Rather, we have used two spin glasses as models for high-dimensional random surfaces. The spin glasses are related by sharing some of their variables, namely the ${\varvec{w}}^{(D)}$, just as the two training objectives in GANs share the discriminator weights. In prior work modeling neural network loss surfaces as spin glasses, the number of spins corresponds to the number of layers in the network, therefore we have chosen *p* spins for $\ell ^{(D)}$ and $p+q$ for $\ell ^{(G)}$, corresponding to *p* layers in the discriminator and *q* layers in the generator, but the generator is only ever seen in the losses composed with the discriminator. One could make other choices of $\ell ^{(D)}$ and $\ell ^{(G)}$ to couple the two glasses and we consider one such example in the appendix Sect. 1.

## Kac-Rice Formulae for Complexity

Training GANs involves jointly minimising the losses of the discriminator and the generator. Therefore, rather than being interested simply in upper-bounding a single spin-glass and counting its stationary points, the complexity of interest comes from jointly upper bounding both $L^{(D)}$ and $L^{(G)}$ and counting points where both are stationary. Using $S^{M}$ to denote the *M*-sphere^2^, we define the complexity

6 $$\begin{aligned} C_{N} =\Bigg |\left\{ {\varvec{w}}^{(D)}\in S^{N_D}, {\varvec{w}}^{(G)}\in S^{N_G } ~:~ \nabla _D L^{(D)}= 0, \nabla _G L^{(G)}= 0, L^{(D)}\in B_D, L^{(G)}\in B_G\right\} \Bigg | \end{aligned}$$

for some Borel sets $B_D, B_G\subset \mathbb {R}$ and where $\nabla _D, \nabla _G$ denote the Riemannian covariate derivatives on the hyperspheres with respect to the discriminator and generator weights respectively. Note:

1. We have chosen to treat the parameters of each network as somewhat separate by placing them on their own hyper-spheres. This reflects the minimax nature of GAN training, where there really are 2 networks being optimised in an adversarial manner rather than one network with some peculiar structure.
2. We could have taken $\nabla = (\nabla _D, \nabla _G)$ and required $\nabla L^{(D)}= \nabla L^{(G)}= 0$ but, as in the previous comment, our choice is more in keeping with the adversarial set-up, with each network seeking to optimize separately its own parameters in spite of the other.
3. We will only be interested in the case $B_D = (-\infty , \sqrt{N} u_D)$ and $B_G= (-\infty , \sqrt{N} u_G)$, for $u_D, u_G\in \mathbb {R}$.

So that the finer structure of local minima and saddle points can be probed, we also define the corresponding complexity with Hessian index prescription

7 $$\begin{aligned} C_{N, k_D, k_G}&=\Bigg |\Bigg \{ {\varvec{w}}^{(D)}\in S^{N_D }, {\varvec{w}}^{(G)}\in S^{N_G } ~:~ \,\nabla _D L^{(D)}= 0, \nabla _G L^{(G)}= 0, L^{(D)}\in B_D, L^{(G)}\in B_G\nonumber \\&\quad i\left( \nabla _D^2 L^{(D)}\right) = k_D, ~i\left( \nabla _G^2 L^{(G)}\right) = k_G \Bigg \}\Bigg |, \end{aligned}$$

where *i*(*M*) is the index of *M* (i.e. the number of negative eigenvalues of *M*). We have chosen to consider the indices of the Hessians $\nabla _D^2 L^{(D)}$ and $\nabla _G^2L^{(G)}$ separately, just as we chose to consider separately vanishing derivatives $\nabla _D L^{(D)}$ and $\nabla _GL^{(G)}$. We believe this choice best reflects the standard training loop of GANs, where each iteration updates the discriminator and generator parameters in separate steps.

To calculate the complexities, we follow the well-trodden route of Kac-Rice formulae as pioneered by [35, 36]. For a fully rigorous treatment, we proceed as in [2, 13].

### Lemma 1

8 $$\begin{aligned} C_N&= \int _{S^{N_D}\times S^{N_G}}\,d{\varvec{w}}^{(G)}d{\varvec{w}}^{(D)}~~\varphi _{\left( \nabla _D L^{(D)}, \nabla _G L^{(G)}\right) }(0)\nonumber \\&\quad \mathbb {E}\left[ \left| \det \left( \begin{array}{cc} \nabla _D^2 L^{(D)}&{} \nabla _{GD} L^{(D)}\\ \nabla _{DG}L^{(G)}&{} \nabla ^2_{G} L^{(G)}\end{array}\right) \right| ~\mid ~ \nabla _GL^{(G)}\right. \nonumber \\&\left. =0, \nabla _DL^{(D)}= 0\right] \mathbbm {1}\left\{ L^{(D)}\in B_D, L^{(G)}\in B_G\right\} \end{aligned}$$

and therefore

9 $$\begin{aligned} C_N&= \int _{S^{N_D}\times S^{N_G}} d{\varvec{w}}^{(G)}d{\varvec{w}}^{(D)}~~\varphi _{\left( \nabla _D L^{(D)}, \nabla _G L^{(G)}\right) }(0)\int _{B_D}dx_D \int _{B_G} dx_G ~ \varphi _{L^{(D)}}(x_D)\varphi _{L^{(G)}}(x_G) \nonumber \\&\quad \mathbb {E}\Bigg [ \left| \det \left( \begin{array}{cc} \nabla _D^2 L^{(D)}&{} \nabla _{GD} L^{(D)}\\ \nabla _{DG}L^{(G)}&{} \nabla ^2_{G} L^{(G)}\end{array}\right) \right| ~\mid ~ \nabla _GL^{(G)}\nonumber \\&=0, \nabla _DL^{(D)}= 0, L^{(D)}= x_D, L^{(G)}= x_G\Bigg ]. \end{aligned}$$

where $\varphi _{(\nabla _D L^{(D)},\nabla _G L^{(G)})}$ is the joint density of $(\nabla _D L^{(D)},\nabla _G L^{(G)})^T$, $\varphi _{L^{(D)}}$ the density of $L^{(D)}$, and $\varphi _{L^{(G)}}$ the density of $L^{(G)}$, all implicitly evaluated at $({\varvec{w}}^{(D)}, {\varvec{w}}^{(G)})$.

### Proof

Routine application of a theorem of [41]. See appendix Sect. 1. $\square$

With Lemma 1 in place, we can now establish the following Kac-Rice expression specialised to our model:

### Lemma 2

For $(N-2)\times (N-2)$ GOE matrix *M* and independent $(N_D - 1)\times (N_D-1)$ GOE matrix $M_1$, define

10 $$\begin{aligned} H\left( x, x_1\right)&\overset{d}{=} bM + b_1\left( \begin{array}{cc} M_1 &{} 0 \\ 0 &{} 0 \end{array}\right) - x - x_1\left( \begin{array}{cc} I_{N_D} &{} 0 \\ 0 &{} 0 \end{array}\right) . \end{aligned}$$

For $$u_G, u_D\in \mathbb {R}$$, define

11 $$\begin{aligned} B = \left\{ (x, x_1)\in \mathbb {R}^2 ~:~ x\le \frac{1}{\sqrt{2}}(p+q)2^{p+q} u_G, ~~ x_1 \ge -(p+q)^{-1} 2^{-(p+q)}p x - \frac{p}{\sqrt{2}}u_D\right\} . \end{aligned}$$

Define the constant

12 $$\begin{aligned} K_N =\omega _{\kappa N}\omega _{\kappa 'N} (2(N-2))^{\frac{N-2}{2}} (2\pi )^{-\frac{N-2}{2}}\left( p + \sigma _z^22^{p+1}(p+q)\right) ^{-\frac{\kappa N-1}{2}} \left( \sigma _z^2 2^{p+q} (p+q)\right) ^{-\frac{\kappa ' N-1}{2}} \end{aligned}$$

where the variances are

13 $$\begin{aligned} s^2 = \frac{1}{2}\sigma _z^2(p+q)^2 2^{3(p+q)}, ~~~ s_1^2 = \frac{p^2}{2}. \end{aligned}$$

and $\omega _N = \frac{2\pi ^{N/2}}{\varGamma (N/2)}$ is the surface area of the *N* sphere. The expected complexity $C_N$ is then

14 $$\begin{aligned} \mathbb {E} C_N = K_N \int _B \sqrt{\frac{N}{2\pi s^2}}e^{-\frac{N}{2s^2}x^2}dx ~ \sqrt{\frac{N}{2\pi s_1^2}} e^{ -\frac{N}{2s_1^2} x_1^2}dx_1 ~\mathbb {E}|\det H(x, x_1)|. \end{aligned}$$

### Proof

Define the matrix

$$\begin{aligned} \tilde{H} = \left( \begin{array}{cc} \nabla _D^2 L^{(D)}&{} \nabla _{GD} L^{(D)}\\ \nabla _{DG}L^{(G)}&{} \nabla ^2_{G} L^{(G)}\end{array}\right) \end{aligned}$$

appearing in the expression for $C_N$ in Lemma 1. Note that $\tilde{H}$ takes the place of a Hessian (though it is not symmetric). We begin with the distribution of

$$\begin{aligned} \tilde{H} ~ \mid ~ \left\{ (\ell ^{(D)}, \ell ^{(G)}) = \left( x_D, x_G\right) , ~ \left( \nabla _D\ell ^{(D)}, \nabla \ell ^{(G)}\right) = (0, 0)\right\} . \end{aligned}$$

Note that the integrand in (14) is jointly spherically symmetric in both ${\varvec{w}}^{(D)}$ and ${\varvec{w}}^{(G)}$. It is therefore sufficient to consider $\tilde{H}$ in the region of a single point on each sphere. We choose the north poles and coordinate bases on both spheres in the region of their north poles. The remaining calculations are routine Gaussian manipulations which appear in the appendix Sect. 1. One finds

15 $$\begin{aligned} \tilde{H}&\overset{d}{=} \sqrt{2p(p-1)} \left( \begin{array}{cc} \sqrt{N_D -1}M^{(D)}_2 &{} 0 \\ 0 &{} 0 \end{array}\right) \nonumber \\&\quad + \sigma _z\sqrt{2^{p+q+1}(p+q)(p+q-1)} \left( \begin{array}{cc} \sqrt{N_D -1}M^{(D)}_1 &{} -2^{-1/2}G \\ 2^{-1/2} G^T &{} \sqrt{N_G - 1}M^{(G)} \end{array}\right) \nonumber \\&\quad - \sigma _z(p+q)x_G2^{p+q} \left( \begin{array}{cc} -I_{N_D} &{} 0 \\ 0 &{} I_{N_G} \end{array}\right) - px_D\left( \begin{array}{cc} I_{N_D} &{} 0 \\ 0 &{} 0 \end{array}\right) \end{aligned}$$

where $M^{(D)}_1, M^{(D)}_2$ are independent $GOE^{N_D - 1}$ matrices, $M^{(G)}$ is an independent $GOE^{N_G - 1}$ matrix and *G* is an independent $(N_D - 1)\times (N_G - 1)$ Ginibre matrix. Note that the dimensions are $N_D - 1$ and $N_G - 1$ rather $N_D$ and $N_G$. This is simply because the hypersphere $S^{N_D}$ is an $N_D - 1$ dimensional manifold, and similarly $S^{N_G}$.

We can simplify by summing independent Gaussians to obtain

16 $$\begin{aligned} \tilde{H} =&\left( \begin{array}{cc} \sigma _D\sqrt{N_D -1}M^{(D)} &{} -2^{-1/2}\sigma _GG, \\ 2^{-1/2} \sigma _G G^T &{} \sigma _G\sqrt{N_G - 1}M^{(G)} \end{array}\right) \nonumber \\&-\sigma _z(p+q)x_G2^{p+q} \left( \begin{array}{cc} -I_{N_D} &{} 0 \\ 0 &{} I_{N_G} \end{array}\right) - px_D\left( \begin{array}{cc} I_{N_D} &{} 0 \\ 0 &{} 0 \end{array}\right) \end{aligned}$$

where

17 $$\begin{aligned} \sigma _G&= \sigma _z\sqrt{2^{p+q+1}(p+q)(p+q-1)} \end{aligned}$$

18 $$\begin{aligned} \sigma _D&= \sqrt{\sigma _G^2 + 2p(p-1)} \end{aligned}$$

and $M^{(D)}\sim GOE^{N_D - 1}$ is a GOE matrix independent of $M^{(G)}$ and *G*.

There is an alternative reformulation of $\tilde{H}$ that will also be useful. Indeed, because $M^{(D)}_{1,2} \overset{d}{=} -M^{(D)}_{1,2}$, let us write $\tilde{H}$ as

19 $$\begin{aligned} \tilde{H}&= \, \sigma _zJ\left( \sqrt{2^{p+q+1}(p+q)(p+q-1)(N_D + N_G - 2)}M_1 - (p+q)x_G2^{p+q}I\right) \nonumber \\&\quad + \left( \sqrt{2p(p-1)(N_D - 1)}\left( \begin{array}{cc} M_2 &{} 0 \\ 0 &{} 0 \end{array}\right) - px_D\left( \begin{array}{cc} I_{N_D} &{} 0 \\ 0 &{} 0 \end{array}\right) \right) \nonumber \\&\overset{d}{=} \, J\Bigg [ \sigma _z\sqrt{2^{p+q+1}(p+q)(p+q-1)(N_D + N_G - 2)}M_1 - \sigma _z(p+q)x_G2^{p+q}I\nonumber \\&\quad + \sqrt{2p(p-1)(N_D - 1)}\left( \begin{array}{cc} M_2 &{} 0 \\ 0 &{} 0 \end{array}\right) + px_D\left( \begin{array}{cc} I_{N_D} &{} 0 \\ 0 &{} 0 \end{array}\right) \Bigg ] \end{aligned}$$

where $M_1\sim GOE^{N_D + N_G - 2}$ is a GOE matrix of size $N_D + N_G-2$, $M_2\sim GOE^{N_D - 1}$ is an independent GOE matrix of size $N_D-1$ and

20 $$\begin{aligned} J = \left( \begin{array}{cc} -I_{N_D} &{} 0 \\ 0 &{} I_{N_G} \end{array}\right) . \end{aligned}$$

If follows that

21 $$\begin{aligned} |\det \tilde{H}| \overset{d}{=}&\Bigg |\det \Bigg [ \sigma _z\sqrt{2^{p+q+1}(p+q)(p+q-1)(N_D + N_G - 2)}M_1 - \sigma _z(p+q)x_G2^{p+q}I\nonumber \\&+ \sqrt{2p(p-1)(N_D - 1)}\left( \begin{array}{cc} M_2 &{} 0 \\ 0 &{} 0 \end{array}\right) + px_D\left( \begin{array}{cc} I_{N_D} &{} 0 \\ 0 &{} 0 \end{array}\right) \Bigg ]\Bigg |. \end{aligned}$$

Now define the constants

22 $$\begin{aligned} b&= \sqrt{2^{p+q}(p+q)(p+q-1)}\sigma _z, \qquad b_1= \sqrt{p(p-1)\kappa } \end{aligned}$$

23 $$\begin{aligned} x&= \frac{\sigma _z(p+q)2^{p+q}}{\sqrt{N}}x_G, \qquad x_1= -\frac{p}{\sqrt{N}}x_D, \end{aligned}$$

and then we arrive at

24 $$\begin{aligned} |\det \tilde{H}| \overset{d}{=} \left( 2(N-2)\right) ^{\frac{N-2}{2}} |\det H(x, x_1)|. \end{aligned}$$

The variances of $L^{(D)}$ and $L^{(G)}$ derive from those of $\ell ^{(G)}, \ell ^{(D)}$ computed in appendix Sect. 1 (see (139), (143)):

$$\begin{aligned} Var(\ell ^{(D)}) = 1, ~~ Var(\ell ^{(G)}) = 2^{p+q}. \end{aligned}$$

Similarly the density $\varphi _{(\nabla _D L^{(D)},\nabla _G L^{(G)})}$ is found in (155):

$$\begin{aligned} \varphi _{\left( \nabla _D L^{(D)}, \nabla _G L^{(G)}\right) }(0) = (2\pi )^{-\frac{N-2}{2}} \left( p + \sigma _z^22^{p+1}(p+q)\right) ^{-\frac{N_D - 1}{2}} \left( \sigma _z^2 2^{p+q} (p+q)\right) ^{-\frac{N_G-1}{2}}. \end{aligned}$$

We have now collected all the inputs required for Lemma 1. The domain of integration *B* arises from the constraints $L^{(D)} \in (-\infty , \sqrt{N} u_D)$ and $L^{(G)} \in (-\infty , \sqrt{N} u_G)$ and the re-scaled variables (23). This completes the proof. $\square$

We will need the asymptotic behaviour of the constant $K_N$, which we now record in a small lemma.

### Lemma 3

As $$N\rightarrow \infty $$,

25 $$\begin{aligned} K_N \sim 2^{\frac{N}{2}}\pi ^{N/2} \left( \kappa ^{\kappa } \kappa '^{\kappa '}\right) ^{-N/2} \sqrt{\kappa \kappa '}\left( p + \sigma _z^22^{p+1}(p+q)\right) ^{-\frac{\kappa N-1}{2}} \left( \sigma _z^2 2^{p+q} (p+q)\right) ^{-\frac{\kappa ' N-1}{2}} \end{aligned}$$

### Proof

By Stirling’s formula

26 $$\begin{aligned} K_N&\sim 4 \pi ^{N} \left( \frac{4\pi }{\kappa N}\right) ^{-1/2}\left( \frac{4\pi }{\kappa ' N}\right) ^{-1/2}\left( \frac{\kappa N}{2 e}\right) ^{-\kappa N/2} \left( \frac{\kappa ' N}{2 e}\right) ^{-\kappa ' N/2} \left( 2(N-2)\right) ^{\frac{N-2}{2}} \left( 2\pi \right) ^{-\frac{N-2}{2}}\nonumber \\&\quad \left( p + \sigma _z^22^{p+1}(p+q)\right) ^{-\frac{\kappa N-1}{2}} \left( \sigma _z^2 2^{p+q} (p+q)\right) ^{-\frac{\kappa ' N-1}{2}}\nonumber \\&\sim 2^{\frac{N}{2}}\pi ^{N/2} \left( \kappa ^{\kappa } \kappa '^{\kappa '}\right) ^{-N/2} \sqrt{\kappa \kappa '}\left( p + \sigma _z^22^{p+1}(p+q)\right) ^{-\frac{\kappa N-1}{2}} \left( \sigma _z^2 2^{p+q} (p+q)\right) ^{-\frac{\kappa ' N-1}{2}} \end{aligned}$$

where we have used $(N-2)^{\frac{N-2}{2}} = N^{\frac{N-2}{2}} \left( 1- \frac{2}{N}\right) ^{\frac{N-2}{2}} \sim N^{\frac{N-2}{2}} e^{-N/2}.$ $\square$

## Limiting Spectral Density of the Hessian

Our intention now is to compute the the expected complexity $\mathbb {E}C_N$ via the Coulomb gas method. The first step in this calculation is to obtain the limiting spectral density of the random matrix

27 $$\begin{aligned} H' = bM + b_1\left( \begin{array}{cc} M_1 &{} 0 \\ 0 &{} 0\end{array}\right) - x_1\left( \begin{array}{cc} I &{} 0 \\ 0 &{} 0\end{array}\right) , \end{aligned}$$

where, note, $H' = H + xI$ is just a shifted version of *H* as defined in Lemma 2. Here the upper-left block is of dimension $\kappa N$, and the overall dimension is *N*. Let $\mu _{eq}$ be the limiting spectral measure of $H'$ and $\rho _{eq}$ its density. The supersymmetric method provides a way of calculating the expected Stieltjes transforms of $\rho _{eq}$ [37]:

28 $$\begin{aligned} \langle G(z) \rangle&= \frac{1}{N} \frac{\partial }{\partial J}\Bigg |_{J=0} Z(J)\end{aligned}$$

29 $$\begin{aligned} Z(J)&:=\mathbb {E}_{H'} \frac{\det (z - H' + J)}{\det (z - H')}. \end{aligned}$$

Recall that a density and its Stieltjes transform are related by the Stieltjes inversion formula

30 $$\begin{aligned} \rho _{eq}(z) = \frac{1}{\pi }\lim _{\epsilon \rightarrow 0} \Im \langle G(z + i\epsilon )\rangle . \end{aligned}$$

The function *Z*(*J*) can be computed using a supersymmetric representation of the ratio of determinants. Firstly, we recall an elementary result from multivariate calculus, where *M* is a real matrix:

31 $$\begin{aligned} \int \prod _{i=1}^N \frac{d\phi _i d\phi _i^*}{2\pi } e ^{-i\phi ^{\dagger }M\phi } = \frac{1}{\det M}. \end{aligned}$$

By introducing the notion of *Grassmann variables* and *Berezin integration*, we obtain a complimentary expression:

32 $$\begin{aligned} \int \frac{1}{-i} \prod _{i=1}^N {d\chi _i d\chi _i^*} e^{-i\chi ^{\dagger }M\chi } = \det {M}. \end{aligned}$$

Here the $\chi _i, \chi _i^*$ are purely algebraic objects defined by the anti-commutation rule

33 $$\begin{aligned} \chi _i\chi _j = - \chi _j\chi _i, ~~ \forall i,j \end{aligned}$$

and $\chi _i^*$ are separate objects, with the complex conjugation unary operator ${}^*$ defined so that $\left( \chi _i^*\right) ^* = -\chi _i^*,$ and Hermitian conjugation is then defined as usual by $\chi ^{\dagger } = (\chi ^T)^*.$ The set of variables $\{\chi _i, \chi _i^*\}_{i=1}^N$ generate a *graded algebra* over $\mathbb {C}$. Mixed vectors of commuting and anti-commuting variables are called *supervectors*, and they belong to a vector space called *superspace*. The integration symbol $\int d\chi _id\chi ^*$ is defined as a formal algebraic linear operator by the properties

34 $$\begin{aligned} \int d\chi _i = 0, ~~~~~ \int d\chi _i ~\chi _j = \delta _{ij}. \end{aligned}$$

Functions of the the Grassmann variables are defined by their formal power series, e.g.

35 $$\begin{aligned} e^{\chi _i} = 1 + \chi _i + \frac{1}{2}\chi _i^2 + \ldots = 1 + \chi _i \end{aligned}$$

where the termination of the series follows from $\chi _i^2 = 0 ~~ \forall i$, which is an immediate consequence of (33). From this it is apparent that (34), along with (33), is sufficient to define Berezin integration over arbitrary functions of arbitrary combinations of Grassmann variables. Finally we establish our notation for supersymmetric (or *graded*) traces of supermatrices. We will encounter supermatrices of the form

$$\begin{aligned} M = \left( \begin{array}{cc} A &{} B \\ C &{} D \end{array}\right) \end{aligned}$$

where *A*, *D* are square block matrices of commuting variables and *B*, *C* are rectangular block matrices of Grassmann variables. In this case, the graded trace is given by $\text {trg}M = \text {Tr}A - \text {Tr}D$. We refer the reader to [42] for a full introduction to supersymmetric methods.

Using the integral results (31), (32) we can then write

36 $$\begin{aligned} \frac{\det (z - H' + J)}{\det (z - H')}&= \int d\varPsi \exp \left\{ -i\phi ^{\dagger }(z-H') \phi - i \chi ^{\dagger }(z+J - H')\chi \right\} \end{aligned}$$

where the measure is

37 $$\begin{aligned} d\varPsi = 1/-i(2\pi )^N \prod _{t=1}^2 {d\phi [t]d\phi ^{*}[t] d\chi [t] d\chi ^*[t]}, \end{aligned}$$

$\phi$ is a vector of *N* complex commuting variables, $\chi$ and $\chi ^*$ are vectors of *N* Grassmann variables, and we use the [*t*] notation to denote the splitting of each of the vectors into the first $\kappa N$ and last $(1-\kappa )N$ components, as seen in [38]:

38 $$\begin{aligned} \phi = \left( \begin{array}{c} \phi [1] \\ \phi [2]\end{array}\right) . \end{aligned}$$

We then split the quadratic form expressions in (36)

39 $$\begin{aligned}&-\phi ^{\dagger }(z-H') \phi - \chi ^{\dagger }(z+J - H')\chi \nonumber \\&\quad = -\phi [1]^{\dagger }\left( x_1 - b_1M_1\right) \phi [1] -\phi ^{\dagger }(z - bM) \phi - \chi [1]^{\dagger }\left( x_1 - b_1 M_1\right) \chi [1] - \chi ^{\dagger }(z + J - b M)\chi . \end{aligned}$$

Taking the GOE averages is now simple [37, 43]:

40 $$\begin{aligned} \mathbb {E}_M \exp \left\{ -ib\phi ^{\dagger }M \phi - ib\chi ^{\dagger }M\chi \right\}&= \exp \left\{ - \frac{b^2}{4N}\text {trg}Q^2\right\} ,\end{aligned}$$

41 $$\begin{aligned} \mathbb {E}_M \exp \left\{ -ib_1\phi [1]^{\dagger }M_1 \phi [1] - ib_1\chi [1]^{\dagger }M_1\chi [1]\right\}&= \exp \left\{ - \frac{b_1^2}{4\kappa N}\text {trg}Q[1]^2\right\} , \end{aligned}$$

where the supersymmetric matrices are given by

42 $$\begin{aligned} Q = \left( \begin{array}{cc} \phi ^{\dagger }\phi &{} \phi ^{\dagger }\chi \\ \chi ^{\dagger }\phi &{} \chi ^{\dagger }\chi \end{array}\right) , ~~~ Q[1] = \left( \begin{array}{cc} \phi [1]^{\dagger }\phi [1] &{} \phi [1]^{\dagger }\chi [1] \\ \chi [1]^{\dagger }\phi [1] &{} \chi [1]^{\dagger }\chi [1]\end{array}\right) . \end{aligned}$$

Introducing the tensor notation

43 $$\begin{aligned} \psi = \phi \otimes \left( \begin{array}{c} 1 \\ 0 \end{array}\right) + \chi \otimes \left( \begin{array}{c} 0 \\ 1 \end{array}\right) , ~~ \psi [1] = \phi [1] \otimes \left( \begin{array}{c} 1 \\ 0 \end{array}\right) + \chi [1] \otimes \left( \begin{array}{c} 0 \\ 1 \end{array}\right) \end{aligned}$$

and

44 $$\begin{aligned} \zeta = \left( \begin{array}{cc} z &{} 0 \\ 0 &{} z + J\end{array}\right) \end{aligned}$$

we can compactly write

45 $$\begin{aligned} Z(J) = \int d\varPsi \exp \left\{ - \frac{b^2}{4N} \text {trg}Q^2 - \frac{b_1^2}{4\kappa N} \text {trg}Q[1]^2 - i \psi [1]^{\dagger }\psi [1]x_1 - i \psi ^{\dagger }\zeta \psi \right\} . \end{aligned}$$

We now perform two Hubbard-Stratonovich transformations [37]

46 $$\begin{aligned} Z(J)&= \int d\varPsi d\sigma d\sigma [1]\exp \left\{ - \frac{N}{b^2} \text {trg}\sigma ^2 - \frac{\kappa N}{b_1^2} \text {trg}\sigma [1]^2\right. \nonumber \\&\quad \left. - i \psi [1]^{\dagger }(x_1 + \sigma [1])\psi [1] - i \psi ^{\dagger }(\sigma + \zeta )\psi \right\} , \end{aligned}$$

where $\sigma$ and $\sigma [1]$ inherit their form from *Q*, *Q*[1]

47 $$\begin{aligned} \sigma = \left( \begin{array}{cc} \sigma _{BB}&{} \sigma _{BF} \\ \sigma _{FB} &{} i\sigma _{FF}\end{array}\right) , ~~~ \sigma [1] = \left( \begin{array}{cc} \sigma _{BB}[1] &{} \sigma _{BF}[1] \\ \sigma _{FB}[1] &{} i\sigma _{FF}[1]\end{array}\right) \end{aligned}$$

with $\sigma _{BB}, \sigma _{FF}, \sigma _{BB}[1], \sigma _{FF}[1]$ real commuting variables, and $\sigma _{BF}, \sigma _{FB}, \sigma _{BF}[1], \sigma _{FB}[1]$ Grassmanns; the factor *i* is introduced to ensure convergence. Integrating out over $d\varPsi$ is now a straightforward Gaussian integral in superspace, giving

48 $$\begin{aligned} Z(J)&= \int d\varPsi d\sigma d\sigma [1] \exp \left\{ - \frac{N}{b^2} \text {trg}\sigma ^2 - \frac{\kappa N}{b_1^2} \text {trg}\sigma [1]^2\right. \nonumber \\&\quad \left. - i \psi [1]^{\dagger }\left( x_1 + \zeta + \sigma + \sigma [1]\right) \psi [1] - i \psi [2]^{\dagger }(\sigma + \zeta )\psi [2]\right\} \nonumber \\&= \int d\sigma d\sigma [1] \exp \left\{ - \frac{N}{b^2} \text {trg}\sigma ^2 - \frac{\kappa N}{b_1^2} \text {trg}\sigma [1]^2\right. \nonumber \\&\quad \left. - \kappa N\text {trg}\log (x_1 + \zeta + \sigma + \sigma [1]) - \kappa ' N \text {trg}\log (\sigma + \zeta )\right\} \nonumber \\&=\int d\sigma d\sigma [1] \exp \left\{ - \frac{N}{b^2} \text {trg}(\sigma - \zeta )^2 - \frac{\kappa N}{b_1^2} \text {trg}\sigma [1]^2\right. \nonumber \\&\quad \left. - \kappa N\text {trg}\log \left( x_1 + \sigma + \sigma [1]\right) - \kappa ' N \text {trg}\log \sigma \right\} . \end{aligned}$$

Recalling the definition of $\zeta$, we have

49 $$\begin{aligned} \text {trg}(\sigma - \zeta )^2 = (\sigma _{BB} - z)^2 - (i\sigma _{FF} - z- J)^2 \end{aligned}$$

and so one immediately obtains

50 $$\begin{aligned} \frac{1}{N}\frac{\partial }{\partial J}\Bigg |_{J=0} Z(J)&= \frac{2}{b^2}\int d\sigma d\sigma [1] (z - i\sigma _{FF}) \exp \Bigg \{ -\frac{N}{b^2} \text {trg}(\sigma - z)^2 - \frac{\kappa N}{b_1^2} \text {trg}\sigma [1]^2\nonumber \\&\quad -\kappa N\text {trg}\log \left( x_1 + \sigma + \sigma [1]\right) - \kappa ' N \text {trg}\log \sigma \Bigg \}\nonumber \\&= \frac{2}{b^2}\int d\sigma d\sigma [1] (z - i\sigma _{FF}) \exp \Bigg \{ -\frac{N}{b^2} \text {trg}\sigma ^2 - \frac{\kappa N}{b_1^2} \text {trg}\sigma [1]^2 \nonumber \\&\quad -\kappa N\text {trg}\log \left( x_1 + z + \sigma + \sigma [1]\right) - \kappa ' N \text {trg}\log ( z+\sigma )\Bigg \} \end{aligned}$$

To obtain the limiting spectral density (LSD), or rather its Stieltjes transform, one must find the leading order term in the $N\rightarrow \infty$ expansion for (50). This can be done by using the saddle point method on the $\sigma ,\sigma [1]$ manifolds. We know that the contents of the exponential must vanish at the saddle point, since the LSD is $\mathcal {O}(1)$, so we in fact need only compute $\sigma _{FF}$ at the saddle point. We can diagonalise $\sigma$ within the integrand of (50) and absorb the diagonalising graded *U*(1/1) matrix into $\sigma [1]$. The resulting saddle point equations for the off-diagonal entries of the new (rotated) $\sigma [1]$ dummy variable are trivial and immediately give that $\sigma [1]$ is also diagonal at the saddle point. The saddle point equations are then

51 $$\begin{aligned}&\frac{2}{b_1^2}\sigma _{BB}[1] + \frac{1}{\sigma _{BB}[1] + \sigma _{BB} + x_1 + z} = 0 \end{aligned}$$

52 $$\begin{aligned}&\frac{2}{b^2}\sigma _{BB} + \frac{\kappa }{\sigma _{BB}[1] + \sigma _{BB} + x_1 + z} + \frac{\kappa '}{\sigma _{BB} + x} = 0 \end{aligned}$$

53 $$\begin{aligned}&\frac{2}{b_1^2}\sigma _{FF}[1] - \frac{1}{\sigma _{FF}[1] + \sigma _{FF} - ix_1 - iz} = 0 \end{aligned}$$

54 $$\begin{aligned}&\frac{2}{b^2}\sigma _{FF} - \frac{\kappa }{\sigma _{FF}[1] + \sigma _{FF} - ix_1 -iz} - \frac{\kappa '}{\sigma _{FF} - iz} = 0. \end{aligned}$$

(53) and (54) combine to give an explicit expression for $\sigma _{FF}[1]$:

55 $$\begin{aligned} \sigma _{FF}[1] = \frac{b_1^2}{2\kappa }\left( \frac{2}{b^2}\sigma _{FF} - \kappa '\left( \sigma _{FF} - iz\right) ^{-1}\right) . \end{aligned}$$

With a view to simplifying the numerical solution of the coming quartic, we define $t = i(\sigma _{FF} - iz)$ and then a line of manipulation with (54) and (55) gives

56 $$\begin{aligned} \left( t^2 - zt - \kappa ' b^2\right) \left( (1 + \kappa ^{-1}b^{-2} b_1^2)t^2 - \left( \kappa ^{-1}b_1^2 b^{-2}z - x_1\right) t - \kappa '\kappa ^{-1}b_1^2\right) + b^2\kappa t^2= 0. \end{aligned}$$

By solving (56) numerically for fixed values of $\kappa , b, b_1, x_1$, we can obtain the four solutions $t_1(z), t_2(z), t_3(z), t_4(z)$. These four solution functions arise from choices of branch for $(z, x_1)\in \mathbb {C}^2$ and determining the correct branch directly is highly non-trivial. However, for any $z\in \mathbb {R}$, at most one of the $t_i$ will lead to a positive LSD, which gives a simple way to compute $\rho _{eq}$ numerically using (30) and (50):

57 $$\begin{aligned} \rho _{eq}(z) = \max _i\left\{ -\frac{2}{b^2\pi } \Im t_i(z)\right\} . \end{aligned}$$

**Fig. 1:**
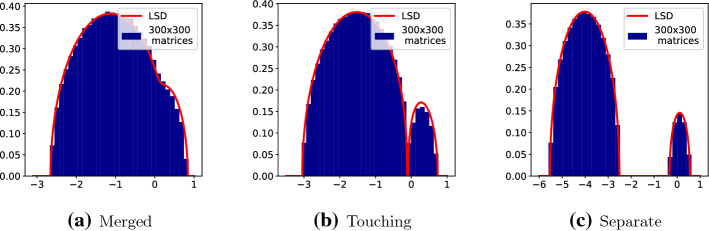
Example spectra of $H'$ showing empirical spectra from 100 $300\times 300$ matrices and the corresponding LSDs computed from (56). Here $b=b_1=1$, $\kappa =0.9$, $\sigma _z$=1 and $x_1$ is varied to give the three different behaviours

Plots generated using (57) and eigendecompositions of matrices sampled from the distribution of $H'$ are given in Fig. 1 and show good agreement between the two. Note the three different forms: single component support, two component support and the transition point between the two, according to the various parameters. In these plots, the larger lobes on the left correspond to the upper left block, which is much larger than the lower-right block (since $\kappa =0.9$ here). One can see this by considering large $x_1$, for which there must be a body of eigenvalues in the region of $-x_1$ owing to the upper left block. Since $x_1$ only features in the upper-left block, not all of the eigenvalues can be located around $-x_1$, and the remainder are found in the other lobe of the density which is around 0 in Fig.  1.

## The Asymptotic Complexity

In the previous section, we have found the equilibrium measure, $\mu _{eq}$, of the ensemble of random matrices

58 $$\begin{aligned} H' = bM + b_1\left( \begin{array}{cc} M_1 &{} 0 \\ 0 &{} 0\end{array}\right) - x_1\left( \begin{array}{cc} I &{} 0 \\ 0 &{} 0\end{array}\right) , ~~ M\sim GOE^N, ~ M_1\sim GOE^{\kappa N}. \end{aligned}$$

The Coulomb gas approximation gives us a method of computing $\mathbb {E} |\det (H'-x)|$:

59 $$\begin{aligned} \mathbb {E} |\det (H'-x)| \approx \exp \left\{ N\int \log |z - x| d\mu _{eq}(z)\right\} . \end{aligned}$$

We have access to the density of $\mu _{eq}$ pointwise (in *x* and $x_1$) numerically, and so (59) is a matter of one-dimensional quadrature. Recalling (14), we then have

60 $$\begin{aligned}&\mathbb {E} C_N \approx K_N'\iint _B dxdx_1 ~ \exp \left\{ -(N-2)\left( \frac{1}{2s^2}x^2 + \frac{1}{2s_1^2} (x_1)^2 - \int \log |z - x| d\mu _{eq}(z) \right) \right\} \nonumber \\&\quad \equiv K_N'\iint _B dxdx_1 ~ e^{-(N-2)\varPhi (x, x_1)} \end{aligned}$$

where

61 $$\begin{aligned} K_N' = K_N \sqrt{\frac{N-2}{2\pi s_1^2}} \sqrt{\frac{N-2}{2\pi s^2}}. \end{aligned}$$

Due to Lemma 3, the constant term has asymptotic form

62 $$\begin{aligned}&\frac{1}{N}\log K_N' \sim \frac{1}{2}\log {2} + \frac{1}{2}\log {\pi } - \frac{\kappa }{2}\log \left( p + \sigma _z^22^{p+q}(p+q)\right) \nonumber \\&\quad - \frac{\kappa '}{2} \log \left( \sigma _z^2(p+q) 2^{p+q}\right) - \frac{\kappa }{2}\log \kappa - \frac{\kappa '}{2}\log \kappa ' \equiv K \end{aligned}$$

We then define the desired $\varTheta (u_D, u_G)$ as

63 $$\begin{aligned} \lim \frac{1}{N} \log \mathbb {E}C_N = \varTheta \left( u_D, u_G\right) \end{aligned}$$

and we have

64 $$\begin{aligned} \varTheta \left( u_D, u_G\right) = K - \min _B \varPhi . \end{aligned}$$

**Fig. 2:**
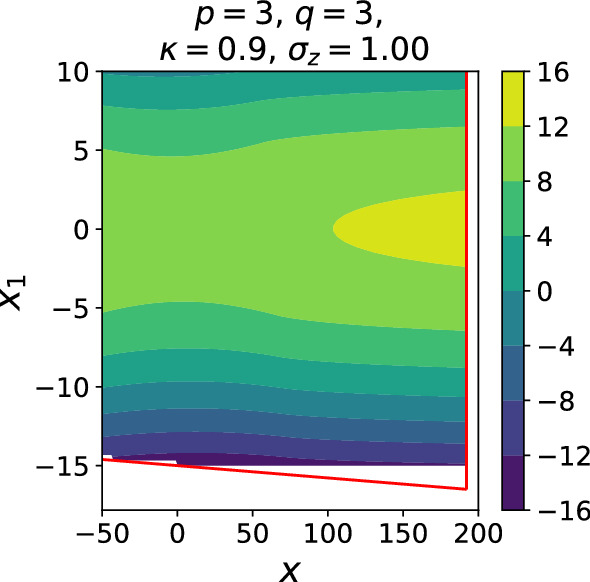
$\varPhi$ for $p=q=3, \sigma _z=1, \kappa =0.9$. Red lines show the boundary of the integration region *B*

**Fig. 3:**
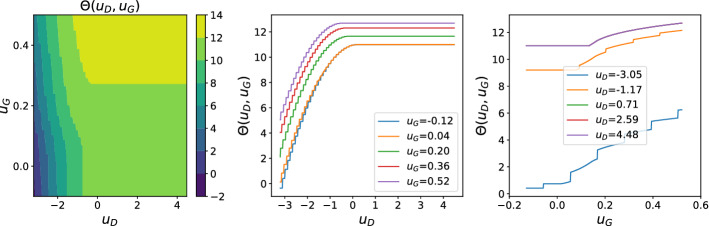
$\varTheta$ and its cross-sections, fixing separately $u_D$ and $u_G$. Here $p=q=3, \sigma _z=1, \kappa =0.9$

Using these numerical methods, we obtain the plot of $\varPhi$ in *B* and a plot of $\varTheta$ for some example $p,q,\sigma _z, \kappa$ values, shown in Figs. 2, 3. Numerically obtaining the maximum of $\varPhi$ on *B* is not as onerous as it may appear, since $-\varPhi$ grows quadratically in $|x|, |x_1|$ at moderate distances from the origin.

**Fig. 4:**
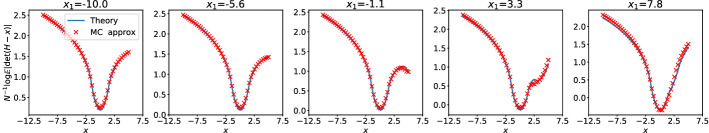
Comparison of (59) and (65), verifying the Coulomb gas approximation numerically. Here $p=q=3, \sigma _z=1, \kappa =0.9$. Sampled matrices for MC approximation are dimension $N=50$, and $n=50$ MC samples have been used

We numerically verify the legitimacy of this Coulomb point approximation with Monte Carlo integration

65 $$\begin{aligned} \mathbb {E}|\det (H'-x)| \approx \frac{1}{n}\sum _{i=1}^n \prod _{j=1}^N |\lambda _j^{(i)} - x|, \end{aligned}$$

where $\lambda ^{(i)}_j$ is the *j*-th eigenvalues of the *i*-th i.i.d. sample from the distribution of $H'$. The results, comparing $N^{-1}\log \mathbb {E}|\det (H'-x)|$ at $N=50$ for a variety of $x,x_1$ are show in Fig. 4. Note the strong agreement even at such modest *N*, however to rigorously substantiate the Coulomb gas approximation in (59), we must prove a concentration result.

### Lemma 4

Let $(H_N)_{N=1}^{\infty }$ be a sequence of random matrices, where for each *N*

66 $$\begin{aligned} H_N \overset{d}{=} bM + b_1\left( \begin{array}{cc} M_1 &{} 0 \\ 0 &{} 0\end{array}\right) - x_1\left( \begin{array}{cc} I &{} 0 \\ 0 &{} 0\end{array}\right) \end{aligned}$$

and $M\sim GOE^N$, $M_1\sim GOE^{\kappa N}$. Let $\mu _N$ be the empirical spectral measure of $H_N$ and say $\mu _N\rightarrow \mu _{eq}$ weakly almost surely. Then for any $(x, x_1)\in \mathbb {R}^2$

67 $$\begin{aligned} \mathbb {E} |\det \left( H_N-xI\right) | = \exp \left\{ N (1 + o(1))\int \log |z - x| d\mu _{eq}(z)\right\} \end{aligned}$$

as $$N\rightarrow \infty $$.

### Proof

We begin by establishing an upper bound. Take any $\beta >0$, then

68 $$\begin{aligned}&\int \log |z-x| d\mu _N(z)\nonumber \\&\quad = \int \log |z-x| \mathbbm {1}\{|x-z|\ge e^{\beta }\} d\mu _N(z) +\int \log |z-x| \mathbbm {1}\{\log |x-z| < \beta \} d\mu _N(z)\nonumber \\&\quad \le \int \log |z-x| \mathbbm {1}\{|x-z|\ge e^{\beta }\} d\mu _N(z) +\int \min (\log |x-z|, \beta ) d\mu _N(z). \end{aligned}$$

Take also any $\alpha >0$, then trivially

69 $$\begin{aligned} \int \min (\log |x-z|, \beta ) d\mu _N(z) \le \int \max (-\alpha , \min (\log |x-z|, \beta )) d\mu _N(z). \end{aligned}$$

Overall we have, for any $\alpha , \beta > 0$,

70 $$\begin{aligned}&\exp \left\{ N\int \log |z-x| d\mu _N(z) \right\} \nonumber \\&\quad \le \exp \left\{ N\int \log |z-x| \mathbbm {1}\{|x-z|\ge e^{\beta }\} d\mu _N(z) \right\} \nonumber \\&\quad \exp \left\{ N \int \max (-\alpha , \min (\log |x-z|, \beta )) d\mu _N(z) \right\} . \end{aligned}$$

Thence an application of Hölder’s inequality gives

71 $$\begin{aligned} \mathbb {E}|\det (H_N-xI)|&= \mathbb {E}\left[ \exp \left\{ N\int \log |z-x| d\mu _N(z)\right\} \right] \nonumber \\&\le \underbrace{\left( \mathbb {E}\left[ \exp \left\{ 2N\int \max \left( -\alpha , \min \left( \log |x-z|, \beta \right) \right) d\mu _N(z)\right\} \right] \right) ^{1/2}}_{A_N}\nonumber \\&\quad \underbrace{\left( \mathbb {E}\left[ \exp \left\{ 2N\int \log |x-z|\mathbbm {1}\{|x-z|\ge e^{\beta }\}d\mu _N(z)\right\} \right] \right) ^{1/2}}_{B_N}. \end{aligned}$$

Considering $B_N$, we have

72 $$\begin{aligned} \log |x-z| \mathbbm {1}\{|x-z| \ge e^{\beta }\} \le |x-z|^{1/2}\mathbbm {1}\{|x-z| \ge e^{\beta }\} \le e^{-\beta /2}|x-z| \end{aligned}$$

and so

73 $$\begin{aligned} \mathbb {E}\left[ \exp \left\{ 2N\int \log |x-z|\mathbbm {1}\{|x-z|\ge e^{\beta }\}\right\} \right]&\le \mathbb {E}\left[ \exp \left\{ 2N e^{-\beta /2} \frac{\text {Tr}|H_N - xI|}{N}\right\} \right] \nonumber \\&= \mathbb {E}\left[ \exp \left\{ 2e^{-\beta /2} \text {Tr}|H_N - xI|\right\} \right] . \end{aligned}$$

The entries of $H_N$ are Gaussians with variance $\frac{1}{N}b^2, \frac{1}{2N}b^2, \frac{1}{N}(b^2 + b_1^2)$ or $\frac{1}{2N}(b^2+b_1^2)$ and all the diagonal and upper diagonal entries are independent. All of these variances are $\mathcal {O}(N^{-1})$, so

74 $$\begin{aligned} |H_N - x|_{ij} \le |x| + |x_1| + \mathcal {O}(N^{-1/2})|X_{ij}| \end{aligned}$$

where the $X_{ij}$ are i.i.d. standard Gaussians for $i\le j$. It follows that

75 $$\begin{aligned} \mathbb {E}\left[ \exp \left\{ 2e^{-\frac{\beta }{2}} \text {Tr}|H_N- xI|\right\} \right]&\le e^{2e^{-\frac{\beta }{2}}N(|x| + |x_1|)}\mathbb {E}_{X\sim \mathcal {N}(0,1)} e^{2e^{-\frac{\beta }{2}}\mathcal {O}(N^{1/2})|X|}. \end{aligned}$$

Elementary calculations give

76 $$\begin{aligned} \mathbb {E}_{X\sim \mathcal {N}(0,1)} e^{c |X|} \le \frac{1}{2}\left( e^{-c^2} + e^{c^2}\right) \le e^{c^2} \end{aligned}$$

and so

77 $$\begin{aligned} \mathbb {E}\left[ \exp \left\{ 2e^{-\frac{\beta }{2}} \text {Tr}|H_N- xI|\right\} \right]&\le e^{2e^{-\frac{\beta }{2}}N(|x| + |x_1|)} e^{4e^{-\beta } \mathcal {O}(N)}\nonumber \\&= \exp \left\{ 2N\left( e^{-\frac{\beta }{2}}(|x| + |x_1|) + e^{-\beta }\mathcal {O}(1)\right) \right\} \end{aligned}$$

thus when we take $\beta \rightarrow \infty$, we have $B_N \le e^{o(N)}$.

Considering $A_N$, it is sufficient now to show

78 $$\begin{aligned} \mathbb {E}\left[ \exp \left\{ 2N \int f(z) d\mu _N(z)\right\} \right] = \exp \left\{ 2N\left( \int f(z) d\mu _{eq}(z) + o(1)\right) \right\} \end{aligned}$$

where $f(z) = 2\max \left( \min (\log |x-z|, \beta ), -\alpha \right)$, a continuous and bounded function. For any $\epsilon >0$, we have

79 $$\begin{aligned}&\mathbb {E}\left[ \exp \left\{ 2N\int f(z) d\mu _N(z)\right\} \right] \nonumber \\&\quad \le \exp \left\{ 2N\left( \int f(z) d\mu _{eq}(z) + \epsilon \right) \right\} + e^{2N||f||_{\infty }}\mathbb {P}\left( \int f(z) d\mu _N(z) \ge \int f(z)d\mu _{eq}(z) + \epsilon \right) . \end{aligned}$$

The entries of $H_N$ are Gaussian with $\mathcal {O}(N^{-1})$ variance and so obey a log-Sobolev inequality as required by Theorem 1.5 from [44]. The constant, *c*, in the inequality is independent of $N, x, x_1$, so we need not compute it exactly. The theorem from [44] then gives

80 $$\begin{aligned} \mathbb {P}\left( \int f(z) d\mu _N(z) \ge \int f(z)d\mu _{eq}(z) + \epsilon \right) \le \exp \left\{ -\frac{N^2}{8c}\epsilon ^2\right\} . \end{aligned}$$

We have shown

81 $$\begin{aligned} \mathbb {E}|\det \left( H_N - xI\right) |&\le A_NB_N \le \exp \left\{ N(1 + o(1))\left( \int f(z)d\mu _{eq}(z)\right) \right\} \nonumber \\&\le \exp \left\{ N(1 + o(1))\left( \int \log |x-z|d\mu _{eq}(z)\right) \right\} . \end{aligned}$$

We now need to establish a complimentary lower bound to complete the proof. By Jensen’s inequality

82 $$\begin{aligned} \mathbb {E}|\det \left( H_N-x\right) |&\ge \exp \left( N\mathbb {E}\left[ \int \log |z-x| d\mu _N(z)\right] \right) \nonumber \\&\ge \exp \left( N\mathbb {E}\left[ \int \max \left( -\alpha , \log |z-x|\right) d\mu _N(z)\right] \right) \nonumber \\&\quad \exp \left( N\mathbb {E}\left[ \int \log |z-x| \mathbbm {1}\{|z-x| \le e^{-\alpha }\}d\mu _N(z)\right] \right) \nonumber \\&\ge \exp \left( N\mathbb {E}\left[ \int \min \left( \beta , \max \left( -\alpha , \log |z-x|\right) \right) d\mu _N(z)\right] \right) \nonumber \\&\quad \exp \left( N\mathbb {E}\left[ \int \log |z-x| \mathbbm {1}\{|z-x| \le e^{-\alpha }\}d\mu _N(z)\right] \right) \end{aligned}$$

for any $\alpha , \beta >0$. Convergence in law of $\mu _N$ to $\mu _{eq}$ and the dominated convergence theorem give

83 $$\begin{aligned} \exp \left( N\mathbb {E}\left[ \int \min \left( \beta , \max \left( -\alpha , \log |z-x|\right) \right) d\mu _N(z)\right] \right) \ge \exp \left\{ N \left( \int \log |x-z| d\mu _{eq}(z) + o(1)\right) \right\} \end{aligned}$$

for large enough $\beta$, because $\mu _{eq}$ has compact support. It remains to show that the expectation inside the exponent in the second term of (82) converges to zero uniformly in *N* in the limit $\alpha \rightarrow \infty$.

By (30), it is sufficient to consider $\langle G_N(z)\rangle$, which is computed via (50). Let us define the function $\varPsi$ so that

84 $$\begin{aligned} \langle G_N(z) \rangle = \frac{2}{b^2} \int d\sigma d\sigma [1] \left( z-i\sigma _{FF}\right) e^{-N\varPsi (\sigma , \sigma [1])}. \end{aligned}$$

Henceforth, $\sigma _{FF}^*, \sigma _{FF}[1]^*, \sigma _{BB}^*, \sigma _{BB}[1]^*$ are the solution to the saddle point equations (51–54) and $\tilde{\sigma }_{FF}, \tilde{\sigma }_{FF}[1], \tilde{\sigma }_{BB}, \tilde{\sigma }_{BB}[1]$ are integration variables. Around the saddle point

85 $$\begin{aligned} z - i\sigma _{FF} = z - i\sigma _{FF}^* - iN^{-\frac{1}{r}}\tilde{\sigma }_{FF} \end{aligned}$$

for some $r\ge 2$. We use the notation ${\varvec{\sigma }}$ for $(\sigma _{BB}, \sigma _{BB}[1], \sigma _{FF}, \sigma _{FF}[1])$ and similarly ${\varvec{\sigma }}_{BB}, {\varvec{\sigma }}_{FF}$. A superscript asterisk on $\varPsi$ or any of its derivatives is short hand for evaluation at the saddle point. While the Hessian of $\varPsi$ may not in general vanish at the saddle point,

86 $$\begin{aligned} \int d\tilde{\sigma }d\tilde{\sigma }[1] \tilde{\sigma }_{FF} e^{-N \tilde{{\varvec{\sigma }}}^T \nabla ^2 \varPsi ^* \tilde{{\varvec{\sigma }}}} = 0 \end{aligned}$$

and so we must go to at least the cubic term in the expansion of $\varPsi$ around the saddle point, i.e.

87 $$\begin{aligned} \langle G_N(z) \rangle = G(z) - \frac{2i}{b^2 N^{5/3}}\underbrace{\int _{-\infty }^{\infty } d\tilde{{\varvec{\sigma }}}_{BB} d\tilde{{\varvec{\sigma }}}_{FF} \tilde{\sigma }_{FF} e^{-\frac{1}{6} \tilde{\sigma }^i \tilde{\sigma }^j \tilde{\sigma }^k \partial _{ijk}\varPsi ^*}}_{E(z; x_1)} + \text { exponentially smaller terms}. \end{aligned}$$

The bosonic (BB) and fermionic (FF) coordinates do not interact, so we can consider derivatives of $\varPhi$ as block tensors. Simple differentiation gives

88 $$\begin{aligned} (\nabla \varPsi )_B&= \left( \begin{array}{c} \frac{2}{b^2}\sigma _{BB} - \kappa \left( \sigma _{BB} + \sigma _{BB}[1] + z + x_1\right) ^{-1} - \kappa '\left( \sigma _{BB} + z\right) ^{-1} \\ \frac{2}{b_1^2}\sigma _{BB}[1] - \left( \sigma _{BB} + \sigma _{BB}[1] + z + x_1\right) ^{-1}\end{array}\right) \nonumber \\&\quad \implies \left( \nabla ^2\varPsi \right) _B\nonumber \\&\quad = \left( \begin{array}{cc} \kappa \left( \sigma _{BB} + \sigma _{BB}[1] + z + x_1\right) ^{-2} + \kappa '\left( \sigma _{BB} + z\right) ^{-2} &{} \kappa \left( \sigma _{BB} + \sigma _{BB}[1] + z + x_1\right) ^{-2}\\ \left( \sigma _{BB} + \sigma _{BB}[1] + z + x_1\right) ^{-2} &{} \left( \sigma _{BB} + \sigma _{BB}[1] + z + x_1\right) ^{-2} \end{array}\right) \end{aligned}$$

89 $$\begin{aligned}&\quad \implies \left( \nabla ^3\varPsi \right) _B^* = \left( \left( \begin{array}{cc} A_B\kappa + B_B\kappa ' &{} A_B\kappa \\ A_B &{} A_B \end{array}\right) , A_B\left( \begin{array}{cc} \kappa &{} \kappa \\ 1 &{} 1 \end{array}\right) \right) , \end{aligned}$$

where

90 $$\begin{aligned} A_B = -\frac{2}{\left( \sigma _{BB}^* + \sigma _{BB}^*[1] + z + x_1\right) ^3}, ~~~ B_B= -\frac{2}{\left( \sigma _{BB}^* + z\right) ^3}. \end{aligned}$$

$(\nabla ^3\varPsi )_F^*$ follows similarly with

91 $$\begin{aligned} A_F = -\frac{2}{\left( \sigma _{FF}^* + \sigma _{FF}^*[1] - iz - ix_1\right) ^3}, ~~~ B_F= -\frac{2}{\left( \sigma _{FF}^* - iz\right) ^3}. \end{aligned}$$

By the saddle point equations (51)–(54) we have

92 $$\begin{aligned} A_B&= 2\left( \sigma _{BB}[1]^*\right) ^3, ~~ B_B = \frac{2}{(\kappa ')^3}\left( \frac{2\kappa }{b_1^2}\sigma _{BB}[1]^* - \frac{2}{b^2} \sigma _{BB}^*\right) ^3 \end{aligned}$$

93 $$\begin{aligned} A_F&= 2\left( \sigma _{FF}[1]^*\right) ^3, ~~ B_F = \frac{2}{(\kappa ')^3}\left( \frac{2\kappa }{b_1^2}\sigma _{FF}[1]^* - \frac{2}{b^2} \sigma _{FF}^*\right) ^3. \end{aligned}$$

Let $$\xi _1= \tilde{\sigma }_{BB}, \xi _2 =\tilde{\sigma }_{BB}[1]$$. Then

94 $$\begin{aligned} \left( \tilde{\sigma }^i \tilde{\sigma }^j \tilde{\sigma }^k \partial _{ijk}\varPhi ^*\right) _B&= \left( A_B\kappa + B_B\kappa '\right) \xi _1^3 + A_B(2\kappa +1 ) \xi _1^2\xi _2[1] + A_B(\kappa +2 ) \xi _1\xi _2^2 + A_B\xi _2^3\nonumber \\&= A_B\left[ \xi _2^3 + (2\kappa +1 )\xi _2\xi _1^2 +(2+ \kappa )\xi _1\xi _2^2 + C\xi _1^3\right] + \left( B_B\kappa ' + A_B\kappa - CA_B\right) \xi _1^3 \end{aligned}$$

for any *C*. Let $\xi _1 = a_1\xi _1'$ and then choose $C = a_1^{-3}$ and $a_1 = (2+\kappa )(2\kappa + 1)^{-1}$ to give

95 $$\begin{aligned} \left( \tilde{\sigma }^i \tilde{\sigma }^j \tilde{\sigma }^k \partial _{ijk}\varPhi ^*\right) _B&= A_B\left( \xi _1' + \xi _2\right) ^3 + \left( B_B\kappa ' + A_B\kappa - CA_B\right) a_1^3(\xi _1')^3 \equiv A_B\eta ^3 + D_B\xi ^3 \end{aligned}$$

with $\eta = \xi _1' + \xi _2$, $\xi =\xi _1'$, $D_B=B_B\kappa ' + A_B\kappa - a_1^{-3}A_B$. The expressions for $( \tilde{\sigma }^i \tilde{\sigma }^j \tilde{\sigma }^k \partial _{ijk}\varPhi ^*)_F$ follow identically. We thus have

96 $$\begin{aligned} E\left( z;x_1\right) \propto \left( \int _0^{\infty } d\xi ~ \xi \int _{\xi }^{\infty }d\eta ~ e^{A_F\eta ^3 + D_F\xi ^3}\right) \left( \int _0^{\infty } d\xi ~ \int _{\xi }^{\infty }d\eta ~ e^{A_B\eta ^3 + D_B\xi ^3}\right) \end{aligned}$$

or perhaps with the the integration ranges reversed depending on the signs of $\Re A_F, \Re A_B, \Re D_F, \Re D_B$. We have

97 $$\begin{aligned} |E\left( z; x_1\right) |&\le \left| \int _0^{\infty } d\xi ~ \xi \int _{\xi }^{\infty }d\eta ~ e^{A_F\eta ^3 + D_F\xi ^3}\right| \cdot \left| \int _0^{\infty } d\xi ~ \int _{\xi }^{\infty }d\eta ~ e^{A_B\eta ^3 + D_B\xi ^3}\right| \nonumber \\&\le \int _0^{\infty } d\xi ~ \xi \int _{\xi }^{\infty }d\eta ~| e^{A_F\eta ^3 + D_F\xi ^3}|\cdot \int _0^{\infty } d\xi ~ \int _{\xi }^{\infty }d\eta ~| e^{A_B\eta ^3 + D_B\xi ^3}|\nonumber \\&\le \int _0^{\infty } d\xi ~ \xi \int _{0}^{\infty }d\eta ~| e^{A_F\eta ^3 + D_F\xi ^3}|\cdot \int _0^{\infty } d\xi ~ \int _{0}^{\infty }d\eta ~| e^{A_B\eta ^3 + D_B\xi ^3}|\nonumber \\&\le \left( |\mathfrak {M} D_F|\right) ^{-2/3}\left( |\mathfrak {M} A_F|\right) ^{-1/3} \left( |\mathfrak {M} D_B|\right) ^{-1/3} \left( |\mathfrak {M} A_B|\right) ^{-1/3}\nonumber \\&\quad \left( \int _0^{\infty } e^{-\xi ^3}d\xi \right) ^3 \left( \int _0^{\infty }~ \xi e^{-\xi ^3}d\xi \right) \end{aligned}$$

where we have defined

98 $$\begin{aligned} \mathfrak {M} y = {\left\{ \begin{array}{ll} \Re y ~~ &{}\text {if } \Re y \ne 0, \\ \Im y ~~ &{}\text {if } \Re y = 0. \\ \end{array}\right. } \end{aligned}$$

This last bound follows from a standard Cauchy rotation of integration contour if any of $D_F, A_F, D_B, A_B$ has vanishing real part. (97) is valid for $D_B, A_B, D_F, A_F \ne 0$, but if $D_B=0$ and $A_B\ne 0$, then the preceding calculations are simplified and we still obtain an upper bound but proportional to $(|\mathfrak {M} A_B|)^{-1/3}$. Similarly with $A_B=0$ and $D_B\ne 0$ and similarly for $A_F, D_F$. The only remaining cases are $A_B = D_B =0$ or $A_F = D_F =0$. But recall (93) and (53)–(54). We immediately see that $A_F=D_F$ if and only if $\sigma _{FF}=\sigma _{FF}[1]=0$, which occurs for no finite $z, x_1$. Therefore, for *fixed* $(x, x_1)\in \mathbb {R}^2$, $\alpha > 0$ and any $z\in (x-e^{-\alpha }, x + e^{-\alpha })$

99 $$\begin{aligned} |\mathbb {E}\mu _N(z) - \mu _{eq}(z; x_1) | \lesssim N^{-5/3} C(x_1, |x| + e^{-\alpha }) \end{aligned}$$

where $C(|x_1|, |x| + e^{-\alpha })$ is positive and is decreasing in $\alpha$. Since $\mu _{eq}$ is bounded, it follows that $\mathbb {E}\mu _N$ is bounded, and therefore

100 $$\begin{aligned} \mathbb {E} \int \log |z-x| \mathbbm {1}\{|z-x| \le e^{-\alpha }\} d\mu _N(z) \rightarrow 0 \end{aligned}$$

as $\alpha \rightarrow \infty$ uniformly in *N*, and so the lower bound is completed. $\square$

Equipped with this result, we can now prove the legitimacy of the Coulomb gas approximation in our complexity calculation. The proof will require an elementary intermediate result which has undoubtedly appeared in various places before, but we prove it here anyway for the avoidance of doubt.

### Lemma 5

Let $M_N$ be a random $N\times N$ symmetric real matrix with independent centred Gaussian upper-diagonal and diagonal entries. Suppose that the variances of the entries are bounded above by $cN^{-1}$ for some constant $c>0$. Then there exists some constant $c_e$ such that

101 $$\begin{aligned} \mathbb {E}||M_N||_{\text {max}}^N \lesssim e^{c_eN}. \end{aligned}$$

### Proof

Let $\sigma _{ij}^2$ denote the variance of $M_{ij}$. Then

102 $$\begin{aligned} \mathbb {E}||M||_{max}^N&\le \sum _{i,j} \mathbb {E}|M_{i,j}|^N\nonumber \\&= \sum _{i,j} \mathbb {E}|\mathcal {N}(0, \sigma _{ij}^2)|^N\nonumber \\&= \sum _{i,j} \sigma _{ij}^N \mathbb {E}|\mathcal {N}(0,1)|^N\nonumber \\&\le N^2c^{N/2} N^{-N/2} \mathbb {E}|\mathcal {N}(0,1)|^N. \end{aligned}$$

Simple integration with a change of variables gives

103 $$\begin{aligned} \mathbb {E}|\mathcal {N}(0,1)|^N&= 2^{\frac{N+1}{2}}\varGamma \left( \frac{N+1}{2}\right) \end{aligned}$$

and then, for large enough *N*, Stirling’s formula gives

104 $$\begin{aligned} \mathbb {E}|\mathcal {N}(0,1)|^N&\sim 2^{\frac{N+1}{2}} \sqrt{\pi (N+1)} \left( \frac{N+1}{2e}\right) ^{\frac{N-1}{2}}\nonumber \\&\sim 2\sqrt{\pi } e^{-\frac{N-1}{2}} N^{N/2} \left( \frac{N+1}{N}\right) ^{N/2}\nonumber \\&\sim 2\sqrt{\pi e} N^{N/2}. \end{aligned}$$

So finally

105 $$\begin{aligned} \mathbb {E}||M||_{max}^N&\lesssim N^2c^{N/2} = e^{\frac{1}{2}N\log {c}+ 2\log {N}} \le e^{\left( \frac{1}{2}\log {c} + 2\right) N}, \end{aligned}$$

so defining $c_e = \frac{1}{2}\log {2} + 2$ gives the result. $\square$

### Theorem 1

For any $x_1\in \mathbb {R}$, let $H_N$ be a random $N\times N$ matrix distributed as in the statement of Lemma 4. Then as $N\rightarrow \infty$

106 $$\begin{aligned}&\iint _B dxdx_1 ~ \exp \left\{ -N\left( \frac{1}{2s^2}x^2 + \frac{1}{2s_1^2} (x_1)^2\right) \right\} \mathbb {E}|\det \left( H_N\left( x_1\right) - x\right) |\nonumber \\&\quad = \iint _B dxdx_1 ~ \exp \left\{ -N\left( \frac{1}{2s^2}x^2 + \frac{1}{2s_1^2} (x_1)^2 - \int \log |z - x| d\mu _{eq}(z) + o(1) \right) \right\} +o(1). \end{aligned}$$

### Proof

Let $R > 0$ be some constant, independent of *N*. Introduce the notation $B_{\le R} = B\cap \{{\varvec{z}}\in \mathbb {R}^2 \mid |z|\le R\}$, and then

107 $$\begin{aligned}&\Bigg |\iint _B dxdx_1 ~ \exp \left\{ -N\left( \frac{1}{2s^2}x^2 + \frac{1}{2s_1^2} (x_1)^2\right) \right\} \mathbb {E}|\det (H_N(x_1) - x)|\nonumber \\&\qquad - \iint _{B_{\le R}} dxdx_1 ~ \exp \left\{ -N\left( \frac{1}{2s^2}x^2 + \frac{1}{2s_1^2} (x_1)^2\right) \right\} \mathbb {E}|\det \left( H_N\left( x_1\right) - x\right) |\Bigg |\nonumber \\&\quad \le \iint _{||{\varvec{x}}||\ge R} dxdx_1 ~ \exp \left\{ -N\left( \frac{1}{2s^2}x^2 + \frac{1}{2s_1^2} (x_1)^2\right) \right\} \mathbb {E}|\det \left( H_N\left( x_1\right) - x\right) |. \end{aligned}$$

We have the upper bound (81) of Lemma 4 but this cannot be directly applied to (107) since the bound relies on uniformity in $x, x_1$ which can only be established for bounded $x, x_1$. We use a much cruder bound instead. First, let

108 $$\begin{aligned} J_N = H_N + x_1 \left( \begin{array}{cc} I &{} 0 \\ 0 &{} 0 \end{array}\right) \end{aligned}$$

and then

109 $$\begin{aligned} |\det \left( H_N - xI\right) | \le ||J_N||_{\text {max}}^N \max \{|x|, |x_1|\}^N = ||J_N||_{\text {max}}^N \exp \left( N\max \{\log |x|, \log |x_1|\}\right) . \end{aligned}$$

$J_N$ has centred Gaussian entries with variance $\mathcal {O}(N^{-1})$, so Lemma 5 applies, and we find

110 $$\begin{aligned} \mathbb {E}|\det \left( H_N - xI\right) | \lesssim \exp \left( N\max \{\log |x|, \log |x_1|\}\right) e^{c_e N} \end{aligned}$$

for some constant $c_e>0$ which is independent of $x, x_1$ and *N*, but we need not compute it.

Now we have

111 $$\begin{aligned}&\Bigg |\iint _B dxdx_1 ~ \exp \left\{ -N\left( \frac{1}{2s^2}x^2 + \frac{1}{2s_1^2} (x_1)^2\right) \right\} \mathbb {E}|\det (H_N(x_1) - x)|\nonumber \\&\qquad - \iint _{B_{\le R}} dxdx_1 ~ \exp \left\{ -N\left( \frac{1}{2s^2}x^2 + \frac{1}{2s_1^2} (x_1)^2\right) \right\} \mathbb {E}|\det (H_N(x_1) - x)|\Bigg |\nonumber \\&\quad \lesssim \iint _{||{\varvec{x}}||\ge R} dxdx_1 ~ \exp \left\{ -N\left( \frac{1}{2s^2}x^2 + \frac{1}{2s_1^2} (x_1)^2 - \max \{\log |x|, \log |x_1|\} - c_e\right) \right\} . \end{aligned}$$

But, since $\mu _{eq}$ is bounded and has compact support, we can choose *R* large enough (independent of *N*) so that

112 $$\begin{aligned} \frac{1}{2s^2}x^2 + \frac{1}{2s_1^2} (x_1)^2 - \max \{\log |x|, \log |x_1|\} - c_e> L > 0 \end{aligned}$$

for all $(x, x_1)$ with $\sqrt{x^2 + x_1^2} > R$ and for some fixed *L* independent of *N*. Whence

113 $$\begin{aligned}&\Bigg |\iint _B dxdx_1 ~ \exp \left\{ -N\left( \frac{1}{2s^2}x^2 + \frac{1}{2s_1^2} (x_1)^2\right) \right\} \mathbb {E}|\det (H_N(x_1) - x)|\nonumber \\&\qquad - \iint _{B_{\le R}} dxdx_1 ~ \exp \left\{ -N\left( \frac{1}{2s^2}x^2 + \frac{1}{2s_1^2} (x_1)^2\right) \right\} \mathbb {E}|\det (H_N(x_1) - x)|\Bigg |\nonumber \\&\quad \lesssim N^{-1}e^{-NL} \rightarrow 0 \end{aligned}$$

as $N\rightarrow \infty$. Finally, for $x, x_1$ in $B_{\le R}$, the result of the Lemma 4 holds uniformly in $x, x_1$, so

114 $$\begin{aligned}&\iint _{B_{\le R}} dxdx_1 ~ \exp \left\{ -N\left( \frac{1}{2s^2}x^2 + \frac{1}{2s_1^2} (x_1)^2\right) \right\} \mathbb {E}|\det (H_N(x_1) - x)|\nonumber \\&\quad = \iint _{B_{\le R}} dxdx_1 ~ \exp \left\{ -N\left( \frac{1}{2s^2}x^2 + \frac{1}{2s_1^2} (x_1)^2 - \int \log |z-x| d\mu _{eq}(z; x_1)+ o(1)\right) \right\} . \end{aligned}$$

The result follows from (113), (114) and the triangle inequality. $\square$

### Asymptotic Complexity with Prescribed Hessian Index

Recall the complexity defined in (7):

$$\begin{aligned} C_{N, k_D, k_G}&=\Bigg |\Bigg \{ {\varvec{w}}^{(D)}\in S^{N_D }, {\varvec{w}}^{(G)}\in S^{N_G } ~:~ \,\nabla _D L^{(D)}= 0, \nabla _G L^{(G)}= 0, L^{(D)}\in B_D, L^{(G)}\in B_G\\&\quad i\left( \nabla _D^2 L^{(D)}\right) = k_D, ~i\left( \nabla _G^2 L^{(G)}\right) = k_G \Bigg \}\Bigg |.\qquad \quad \qquad \qquad (7) \end{aligned}$$

The extra Hessian signature conditions in (7) enforce that both generator and discriminator are at low-index saddle points. Our method for computing the complexity $C_N$ in the previous subsection relies on the Coulomb gas approximation applied to the spectrum of $H'$. However, the Hessian index constraints are formulated in the natural Hessian matrix (16), but our spectral calculations proceed from the rewritten form (21). We find however that we can indeed proceed much as in [13]. Recall the key Hessian matrix $\tilde{H}$ given in (16) by

115 $$\begin{aligned} \tilde{H}&= \left( \begin{array}{cc} \sqrt{2(N_D - 1)}\sqrt{b^2 + b_1^2}M^{(D)} &{} -bG \\ b G^T &{} \sqrt{2(N_G-1)}bM^{(G)} \end{array}\right) \nonumber \\&\quad -\sqrt{N-2}x \left( \begin{array}{cc} -I_{N_D} &{} 0 \\ 0 &{} I_{N_G} \end{array}\right) + \sqrt{N-2}x_1\left( \begin{array}{cc} I_{N_D} &{} 0 \\ 0 &{} 0 \end{array}\right) \end{aligned}$$

where $M^{(D)}\sim GOE^{N_D -1}$, $M^{(G)}\sim GOE^{N_G-1}$, *G* is $N_D - 1 \times N_G - 1$ Ginibre, and all are independent. Note that we have used (23) to slightly rewrite (16). We must address the problem of computing

116 $$\begin{aligned}&\mathbb {E}|\det \tilde{H}|\mathbbm {1}\left\{ i\left( \sqrt{\kappa }(1 + \mathcal {O}(N^{-1}))\sqrt{b^2 + b_1^2}M_D + \frac{x+x_1}{\sqrt{2}}\right) \right. \nonumber \\&\quad \left. = k_D, ~ i\left( \sqrt{\kappa '}(1 + \mathcal {O}(N^{-1}))bM_G - \frac{x}{\sqrt{2}}\right) = k_G\right\} . \end{aligned}$$

Indeed, we introduce integration variables ${\varvec{y}}_1, {\varvec{y}}_2, \zeta _1, \zeta _1^*, \zeta _2,\zeta _2^*$, being $(N-2)$-vectors of commuting and anti-commuting variables respectively. Use [*t*] notation to split all vectors into the first $\kappa N -1$ and last $\kappa 'N-1$ components. Let

117 $$\begin{aligned} A[t] = {\varvec{y}}_1{\varvec{y}}_1^T + {\varvec{y}}_2{\varvec{y}}_2^T + \zeta _1\zeta _1^{\dagger } + \zeta _2\zeta _2^{\dagger }. \end{aligned}$$

With these definitions, we have [13]

118 $$\begin{aligned} |\det \tilde{H}|&= (2(N-2))^{\frac{N-2}{2}} \lim _{\epsilon \searrow 0} \int d\varXi \nonumber \\&\quad \exp \left\{ -i\sqrt{\kappa }(1 + \mathcal {O}(N^{-1})) \sqrt{b^2 + b_1^2}\text {Tr}M^{(D)} A[1]-i\sqrt{\kappa '}(1 + \mathcal {O}(N^{-1})) b \text {Tr}M^{(G)} A[2] \right\} \nonumber \\&\quad \exp \{ \mathcal {O}(\epsilon )\}\exp \{\ldots \} \end{aligned}$$

where $d\varXi$ is the normalised measure of the ${\varvec{y}}_1, {\varvec{y}}_2, \zeta _1, \zeta _1^*, \zeta _2,\zeta _2^*$ and the ellipsis represents terms with no dependence on $M^{(D)}$ or $M^{(G)}$, which we need not write down. The crux of the matter is that we must compute

119 $$\begin{aligned}&\mathbb {E}_{M^{(D)}}e^{-i\sqrt{\kappa } \sqrt{b^2 + b_1^2}\text {Tr}M^{(D)} A[1]}\mathbbm {1}\left\{ i\left( M_D + \frac{x + x_1}{\sqrt{\kappa }\sqrt{b^2 + b_1^2}}(1 + \mathcal {O}(N^{-1}))\right) = k_D\right\} , \end{aligned}$$

120 $$\begin{aligned}&\mathbb {E}_{M^{(G)}}e^{-i\sqrt{\kappa '} b \text {Tr}M^{(G)} A[2]}\mathbbm {1}\left\{ i\left( M_G - \frac{x}{\sqrt{\kappa '}b}(1 + \mathcal {O}(N^{-1}))\right) = k_G\right\} , \end{aligned}$$

but [13] has performed exactly these calculations (see around (5.146) therein) and so there exist constants $K^{(D)}_U,K^{(D)}_L, K^{(G)}_U,K^{(G)}_L$ such that

121 $$\begin{aligned}&K^{(D)}_L e^{-Nk_D\kappa (1 + o(1)) I_1(\hat{x}_D; \sqrt{2})} e^{-\frac{1}{2N}(b^2 + b_1^2)\text {Tr}A[1]^2} \nonumber \\&\quad \le \Re \mathbb {E}_{M^{(D)}}e^{-i\sqrt{\kappa } \sqrt{b^2 + b_1^2}\text {Tr}M^{(D)} A[1]}\mathbbm {1}\left\{ i\left( M_D + \frac{x + x_1}{\sqrt{\kappa }\sqrt{b^2 + b_1^2}}(1 + \mathcal {O}(N^{-1}))\right) = k_D\right\} \nonumber \\&\quad \le K^{(D)}_U e^{-Nk_D\kappa (1 + o(1)) I_1(\hat{x}_D; \sqrt{2})} e^{-\frac{1}{2N}(b^2 + b_1^2)\text {Tr}A[1]^2} \end{aligned}$$

and

122 $$\begin{aligned}&K^{(G)}_L e^{-Nk_G\kappa '(1 + o(1)) I_1(\hat{x}_G; \sqrt{2})} e^{-\frac{1}{2N}b^2 \text {Tr}A[2]^2} \nonumber \\&\quad \le \Re \mathbb {E}_{M^{(G)}}e^{-i\sqrt{\kappa '} b\text {Tr}M^{(G)} A[2]}\mathbbm {1}\left\{ i\left( M_G - \frac{x}{\sqrt{\kappa '}b}(1 + \mathcal {O}(N^{-1}))\right) = k_G\right\} \nonumber \\&\quad \le K^{(G)}_U e^{-Nk_G\kappa '(1 + o(1)) I_1(\hat{x}_G; \sqrt{2})} e^{-\frac{1}{2N}b^2 \text {Tr}A[2]^2} \end{aligned}$$

where

123 $$\begin{aligned} \hat{x}_D = -\frac{x + x_1}{\sqrt{\kappa }\sqrt{b^2 + b_1^2}}, ~~ \hat{x}_G = \frac{x}{\sqrt{\kappa '}b}. \end{aligned}$$

Here $I_1$ is the rate function of the largest eigenvalue of the GOE as obtained in [45] and used in [2, 13]:

124 $$\begin{aligned} I_1(u; E) = {\left\{ \begin{array}{ll} \frac{2}{E^2}\int _u^{-E} \sqrt{z^2 - E^2}dz ~ &{}\text { for } u< -E,\\ \frac{2}{E^2}\int _{E}^u \sqrt{z^2 - E^2}dz ~ &{}\text { for } u > E,\\ \infty &{}\text { for } |u| < E. \end{array}\right. } \end{aligned}$$

Note that for $u< -E$

125 $$\begin{aligned} I_1(u; E) = -\frac{u}{E}\sqrt{u^2 - E^2} - \log \left( -u + \sqrt{u^2 - E^2}\right) + \log {E} \end{aligned}$$

and for $u>E$ we simply have $I_1(u; E) = I_1(-u; E)$. Note also that $I_1(ru; E) = I_1(u, E/r).$

We have successfully dealt with the Hessian index indicators inside the expectation, however we need some way of returning to the form of $\tilde{H}$ in (21) so the complexity calculations using the Coulomb gas approach can proceed as before. We can achieve this with inverse Fourier transforms:

126 $$\begin{aligned} e^{-\frac{1}{2N}(b^2 + b_1^2)\text {Tr}A[1]^2}&= \mathbb {E}_{M_D}e^{-i\sqrt{\kappa }\sqrt{b^2 + b_1^2}\text {Tr}M_DA[1]} \end{aligned}$$

127 $$\begin{aligned} e^{-\frac{1}{2N}b^2\text {Tr}A[2]^2}&= \mathbb {E}_{M_G}e^{-i\sqrt{\kappa '}b\text {Tr}M_GA[2]} \end{aligned}$$

from which we obtain

128 $$\begin{aligned}&K_Le^{-Nk_D\kappa (1 + o(1)) I_1(\hat{x}_D; \sqrt{2})} e^{-Nk_G\kappa '(1 + o(1)) I_1(\hat{x}_G; \sqrt{2})}\mathbb {E}|\det \tilde{H}|\nonumber \\&\quad \le \mathbb {E}|\det \tilde{H}|\mathbbm {1}\left\{ i\left( \sqrt{\kappa }(1 + \mathcal {O}(N^{-1}))\sqrt{b^2 + b_1^2}M_D + \frac{x+x_1}{\sqrt{2}}\right) \right. \nonumber \\&\quad \left. = k_D, ~ i\left( \sqrt{\kappa '}(1 + \mathcal {O}(N^{-1}))bM_G - \frac{x}{\sqrt{2}}\right) = k_G\right\} \end{aligned}$$

129 $$\begin{aligned}&\le K_Ue^{-Nk_D\kappa (1 + o(1)) I_1(\hat{x}_D; \sqrt{2})} e^{-Nk_G\kappa '(1 + o(1)) I_1(\hat{x}_G; \sqrt{2})} \mathbb {E}|\det \tilde{H}|. \end{aligned}$$

It follows that

130 $$\begin{aligned}&K_N'\iint _B dx dx_1 e^{-(N-2)\left[ \varPhi (x, x_1) + k_G\kappa ' I_1(x; \sqrt{2\kappa '}b) + k_D\kappa I_1\left( ( - (x + x_1); \sqrt{2\kappa (b^2 + b_1^2)}\right) \right] (1 + o(1))} \nonumber \\&\quad \lesssim C_{N, k_D, k_G} \nonumber \\&\quad \lesssim K_N' \iint _B dx dx_1 e^{-(N-2)\left[ \varPhi (x, x_1) + k_G\kappa ' I_1(x; \sqrt{2\kappa '}b) + k_D\kappa I_1\left( ( - (x + x_1); \sqrt{2\kappa (b^2 + b_1^2)}\right) \right] (1 + o(1))}. \end{aligned}$$

So we see that the relevant exponent in this case is the same as for $C_N$ but with additional GOE eigenvalue large deviation terms, giving the complexity limit

131 $$\begin{aligned} \lim \frac{1}{N} \log \mathbb {E}C_{N, k_D, k_G}&= \varTheta _{k_D, k_G}\left( u_D, u_G\right) \nonumber \\&= K - \min _B \left\{ \varPhi + k_G\kappa ' I_1(x; \sqrt{2\kappa '}b) + k_D\kappa I_1\left( - (x + x_1); \sqrt{2\kappa (b^2 + b_1^2)}\right) \right\} . \end{aligned}$$

**Fig. 5:**
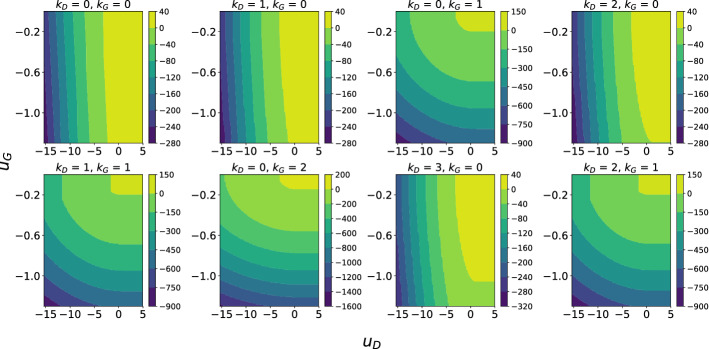
Contour plots of $\varTheta _{k_D, k_G}$ for a few values of $k_D, k_G$. Here $p=q=3, \sigma _z=1, \kappa =0.9$

Plots of $\varTheta _{k_D, k_G}$ for a few values of $k_D, k_G$ are shown in Fig. 5.

#### Remark 2

Recall that the limiting spectral measure of the Hessian displays a transition as the support splits from one component to two, as shown in Fig. 1. Let us comment on the relevance of this feature to the complexity. The spectral measure appears in one place in the above complexity calculations: the Coulomb gas integral $\int d\mu _{eq}(z) \log |z - x|$. The effect of integrating against the measure $\mu _{eq}$ is to smooth out the transition point. In other words, if $\mu _{eq}$ has two components or is at the transition point, one expects to be able to construct another measure $\nu$ supported on a single component such that $\int d\nu (z) \log |z - x| = \int d\mu _{eq}(z) \log |z - x|$. We interpret this to mean that the Coulomb gas integral term does not display any features that can be unambiguously attributed to the transition behaviour of the spectral measure.

## Implications

### Structure of Low-Index Critical Points

**Fig. 6:**
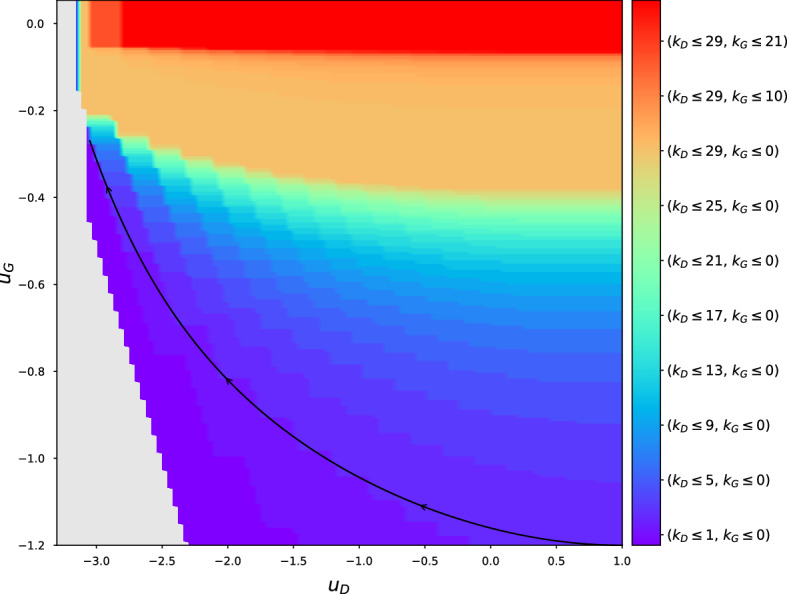
Contours in the $(u_D, u_G)$ plane of the maximum $k_D$ and $k_G$ such that $\varTheta _{k_D, k_G}(u_D, u_G)>0$. $k_D$ results shown with a red colour red scheme, and $k_G$ with blue/green. The grey region on the left lies outside the domain of definition of $\varTheta _{k_D, k_G}$. Here $p=q=3, \sigma _z=1, \kappa =0.9$. The arrow indicates the approximate location of the contour discussed in the main text

We examine the fine structure of the low-index critical points for both spin glasses. [1] used the ‘banded structure’ of low-index critical points to explain the effectiveness of gradient descent in large multi-layer perceptron neural networks. We undertake to uncover the analogous structure in our dual spin-glass model and thence offer explanations for GAN training dynamics with gradient descent. For a range of $(k_D, k_G)$ values, starting at (0, 0), we compute $\varTheta _{k_D, k_G}$ on an appropriate domain. In the $(u_D, u_G)$ plane, we then find the maximum $k_D$, and separately $k_G$, such that $\varTheta _{k_D, k_G}(u_D, u_G)>0$. In the large *N* limit, this procedure reveals the regions in the $(u_D, u_G)$ plane where critical points of each index of the two spin glasses are found. Figure 6 plots these maximum $k_D, k_G$ values as contours on a shared $(u_D, u_G)$ plane. The grey region in the plot clearly shows the ‘ground state’ boundary beyond which no critical points exist. We use some fixed values of the various parameters: $p=q=3, \sigma _z=1, \kappa =0.9$.

These plots reveal, unsurprisingly perhaps, that something resembling the banded structure of [1] is present, with the higher index critical points being limited to higher loss values for each network. The 2-dimensional analogues of the $E_{\infty }$ boundary of [1] are evident in the bunching of the $k_D, k_G$ contours at higher values. There is, however further structure not present in the single spin-glass multi-layer perceptron model. Consider the contour of $k_D = 0$ at the bottom of the full contour plot in Fig. 6. Imagine traversing a path near this contour from right to left (decreasing $u_D$ values); an example path is approximately indicated by a black arrow on the figure. At all points along such a path, the only critical points present are exact local minima for both networks, however the losses range over

(i) low generator loss, high discriminator loss;
(ii) some balance between generator and discriminator loss;
(iii) high generator loss, low discriminator loss.

These three states correspond qualitatively to known GAN phenomena:

(i) discriminator collapses to predicting ‘real’ for all items;
(ii) successfully trained model;
(iii) generator collapses to producing garbage samples which the discriminator trivially identifies.

**Fig. 7:**
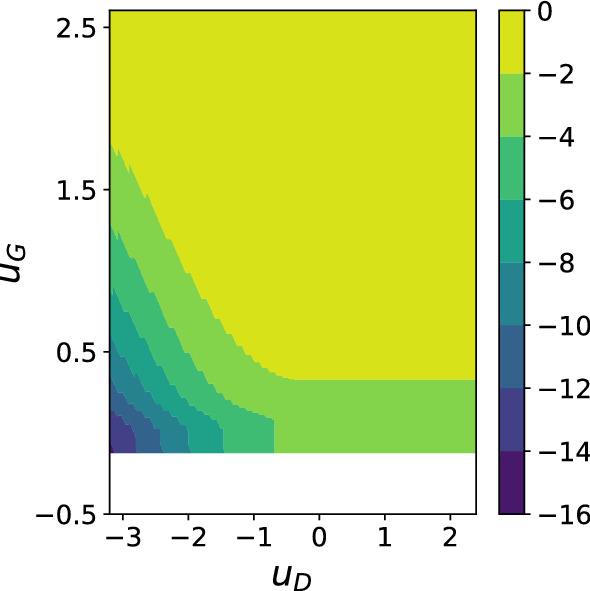
Contour plot of the log ratio quantity given in (132). This is the approximate proportion of minima with both $L_D \le u_D$ and $L_G\le u_G$ out of all points where at at least one of those conditions holds

Overall, the analysis of our model reveals a loss surface that favours convergence to states of low loss for *at least one of the networks*, but not necessarily both. Moreover, our plots of $\varTheta$ and $\varTheta _{k_D, k_G}$ in Figs. 3, 5 demonstrate clearly the competition between the two networks, with the minimum attainable discriminator loss increasing as the generator loss decreases and vice-versa. We thus have a qualitative similarity between the minimax dynamics of real GANs and our model, but also a new two-dimensional banded critical points structure. We can further illuminate the structure by plotting, for each $(u_D, u_G)$, the approximate proportion of minima with both $L_D \le u_D$ and $L_G\le u_G$ out of all points where at at least one of those conditions holds. The expression is

132 $$\begin{aligned} \varTheta \left( u_D, u_G\right) - \max \left\{ \varTheta \left( u_D, \infty \right) , \varTheta \left( \infty , u_G\right) \right\} \end{aligned}$$

which gives the log of the ratio in units of *N*. We show the plot in Fig. 7. Note that, for large *N*, any region of the plot away from a value of zero contains exponentially more bad minima – where one of the networks has collapsed – than good minima, with equilibrium between the networks. The model therefore predicts the existence of good local minima (in the bottom left of Fig. 7) that are effectively inaccessible due to their being exponentially outnumbered by bad local minima.

The structure revealed by our analysis offers the following explanation of large GAN training dynamics with gradient descent:

1. As with single feed-forward networks, the loss surface geometry encourages convergence to globally low values of at least one of the network losses.
2. The same favourable geometry encourages convergence to successful states, where both networks achieve reasonably low loss, but also encourages convergence to failure states, where the generator’s samples are too easily distinguished by the discriminator, or the discriminator has entirely failed thus providing no useful training signal to the generator.

#### Remark 3

A natural question in the context of our analysis of low-index critical points is: do such points reflect the points typically reached by gradient descent algorithms used to train real GANs? There has been much discussion in the literature of the analogous question for single networks and spin glasses [1, 7, 9]. It is not clear how to settle this question in our case, but we believe our model and its low-index critical points give a description of the baseline properties to be expected of high-dimensional adversarial optimisation problems late in the optimisation procedure. In addition, the unstructured random noise present in spin glasses may be more appropriate in our model for GANs than it is for single spin-glass models of single networks, as GAN generators do genuinely contain unstructured latent noise, rather than just the highly-structured data distributions seen on real data.

#### Remark 4

The issue of meta-stability is also worth mentioning. In single spin glasses, the boundary $E_{\infty }$ between fixed index and unbounded index critical points is meta-stable [46, 47]. From the random matrix theory perspective, the $E_{\infty }$ boundary corresponds to the left edge of the Wigner semi-circle [2]. There are *O*(*N*) eigenvalues in any finite interval at the left of the Wigner semi-circle, corresponding to *O*(*N*) Hessian eigenvalues in any neighbourhood around zero. The 2D analogue of the $E_{\infty }$ boundary in our double spin-glass model is expected to possess the same meta-stability: the Wigner semi-circle is replaced by the measure studied in Sect. 4, to which the preceding arguments apply. In the context of deep neural networks, there is a related discussion concerning “wide and flat local optima” of the loss surface, i.e. local optima for which many of the Hessian eigenvalues are close to zero. There are strong indications that deep neural networks converge under gradient-based optimisation to such optima [48–53] and that they are perhaps better for generalisation (i.e. test set loss) than other local optima, however some authors have challenged this view [54–58]. It is beyond the scope of the present work to analyse the role of meta-stability further, however we note that the indications from machine learning are that it is most significant when considering generalisation, however our work simplifies to the case of a single loss rather than separately considering training and test loss.

### Hyperparameter Effects

Our proposed model for GANs includes a few fixed hyperparameters that we expect to control features of the model, namely $\sigma _z$ and $\kappa$. Based on the results of [1, 2, 13], and the form of our analytical results above, we do not expect *p* and *q* (the number of layers in the discriminator and generator) to have any interesting effect beyond $p, q \ge 3$; this is clearly a limitation of the model. We would expect there to exist an optimal value of $\sigma _z$ that would result in minimum loss, in some sense. The effect of $\kappa$ is less clear, though we guess that, in the studied $N\rightarrow \infty$ limit, all $\kappa \in (0, 1)$ are effectively equivalent. Intuitively, choosing $\kappa =0, 1$ corresponds to one network having a negligible number of parameters when compared with the other and we would expect the much larger network to prevail in the minimax game, however our theoretical results above are valid strictly for $\kappa \in (0,1)$.

In the following two subsections we examine effect of $\sigma _z$ and $\kappa$ in our theoretical and in real experiments with a DCGAN [26]. Additional supporting plots are given in the appendix.

#### Effect of Variance Ratio

In the definition of complexity, $u_D$ and $u_G$ are upper bounds on the loss of the discriminator and generator, respectively. We are interested in the region of the $u_D,u_G$ plane such that $\varTheta (u_D, u_G)>0$, this being the region where gradient descent algorithms are expected to become trapped. We therefore investigate the minimum loss such that $\varTheta > 0$, this being, for a given $\sigma _z$, the theoretical minimum loss attainable by the GAN. We consider two natural notions of loss:

1. $$\vartheta _D = \min \{u_D\in \mathbb {R} \mid \exists u_G\in \mathbb {R} ~:~ \varTheta (u_D, u_G) > 0 \} $$;
2. $$\vartheta _G = \min \{u_G\in \mathbb {R} \mid \exists u_D\in \mathbb {R} ~:~ \varTheta (u_D, u_G) > 0 \} $$.

We vary $\sigma _z$ over a range of values in $(10^{-5}, 10^{2})$ and compute $\vartheta _D, \vartheta _G$.

**Fig. 8:**
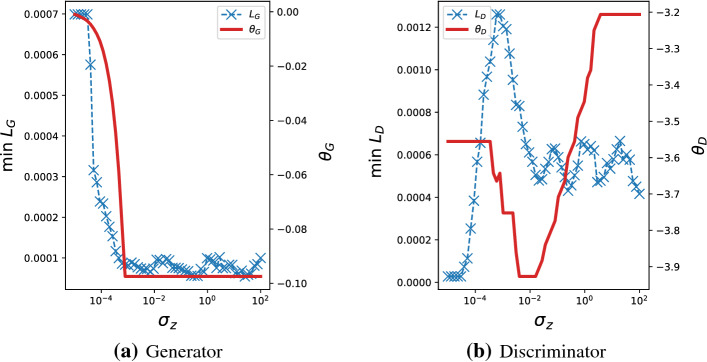
The effect of $\sigma _z$. Comparison of theoretical predictions of minimum possible discriminator and generator losses to observed minimum losses when training DCGAN on CIFAR10. The blue cross-dashed lines show the experimental DCGAN results, and solid red lines show the theoretical results $\theta _G, \theta _D$. $p=q=5$ and $\kappa =0.5$ are used in the theoretical calculations, to best match the DCGAN architecture. $\sigma _z$ is shown on a log-scale

To compare the theoretical predictions of the effect of $\sigma _z$ to real GANs, we perform a simple set of experiments. We use a DCGAN architecture [26] with 5 layers in each network, using the reference PyTorch implementation from [59], however we introduce the generator noise scale $\sigma _z$. That is, the latent input noise vector ${\varvec{z}}$ for the generator is sampled from $\mathcal {N}(0, \sigma _z^2I)$. For a given $\sigma _z$, we train the GANs for 10 epochs on CIFAR10 [60] and record the generator and discriminator losses. For each $\sigma _z$, we repeat the experiment 30 times and average the minimum attained generator and discriminator losses to account for random variations between runs with the same $\sigma _z$. We note that the sample variances of the loss were typically very high, despite the PyTorch random seed being fixed across all runs. We plot the sample means, smoothed with rolling averaging over a short window, in the interest of clearly visualising whatever trends are present. The results are shown in Fig. 8.

There is a striking similarity between the generator plots, with a sharp decline between $\sigma _z=10^{-5}$ and around $10^{-3}$, after which the minimum loss is approximately constant. The picture for the discriminator is less clear. Focusing on the sections $\sigma _z > 10^{-3}$, both plots show a clear minimum, at around $\sigma _z=10^{-1}$ in experiments and $\sigma _z=10^{-2}$ in theory. Note that the scales on the *y*-axes of these plots should not be considered meaningful. Though there is not precise correspondence between the discriminator curves, we claim that both theory and experiment tell the same qualitative story: increasing $\sigma _z$ to at least around $10^{-3}$ gives the lowest theoretical generator loss, and then further increasing to, tentatively, some value in $(10^{-2}, 10^{-1})$ gives the lowest possible discriminator loss at no detriment to the generator.

We are not aware of $\sigma _z$ tuning being widely used in practice for real GANs, rather it is typically taken to be unity. We have chosen this parameter, as it can be directly paralleled in our spin glass model, therefore allowing for the above experimental comparison. Naturally there are other parameters of real GANs that one might wish to study (such as learning rates and batch sizes) however these are much less readily mirrored in the spin glass model and complexity analysis, precluding comparisons between theory and experiment. Nevertheless, the experimental results in Fig. 8 do demonstrate that tuning $\sigma _z$ in real GANs could be of benefit, as $\sigma _z=1$ does not appear to be the optimal value.

#### Effect of Size Ratio

**Fig. 9:**
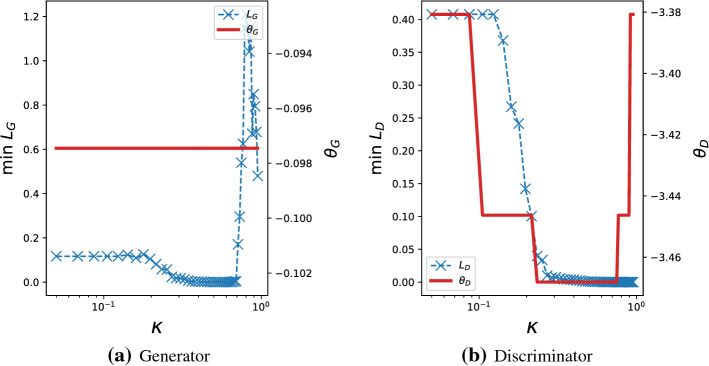
The effect of $\kappa$. Comparison of theoretical predictions of minimum possible discriminator and generator losses to observed minimum losses when training DCGAN on CIFAR10. The blue cross-dashed lines show the experimental DCGAN results, and the solid red show the theoretical results $\vartheta _G, \vartheta _D$. $p=q=5$ and $\sigma _z=1$ are used in the theoretical calculations, to best match the DCGAN architecture

Similarly to the previous section, we can investigate the effect of $\kappa$ using $\vartheta _D, \vartheta _G$ while varying $\kappa$ over (0, 1). To achieve this variation in the DCGAN, we vary the number of convolutional filters in each network. The generator and discriminator are essentially mirror images of each other and the number of filters in each intermediate layer are defined as increasing functions^3^ of some positive integers $n_G, n_D$. We fix $n_D + n_G=128$ and vary $n_D$ to obtain a range of $\kappa$ values, with $\kappa = \frac{n_d}{n_d + n_g}$. The results are shown in Fig. 9.

The theoretical model predicts a a broad range of equivalently optimal $\kappa$ values centred on $\kappa =0.5$ from the perspective of the discriminator loss, and no effect of $\kappa$ on the generator loss. The experimental results similarly show a broad range of equivalently optimal $\kappa$ centred around $\kappa =0.5$, however there appear to be deficiencies in our model, particularly for higher $\kappa$ values. The results of the experiments are intuitively sensible: the generator loss deteriorates for $\kappa$ closer to 1, i.e. when the discriminator has very many more parameters than the generator, and vice-versa for small $\kappa$.

## Conclusions and Outlook

We have contributed a novel model for the study of large neural network gradient descent dynamics with statistical physics techniques, namely an interacting spin-glass model for generative adversarial neural networks. We believe this is the first attempt in the literature to incorporate advanced architectural features of modern neural networks, beyond basic single network multi-layer perceptrons, into such statistical physics style models. We have conducted an asymptotic complexity analysis via Kac-Rice formulae and Random Matrix Theory calculations of the energy surface of this model, acting as a proxy for GAN training loss surfaces of large networks. Our analysis has revealed a banded critical point structure as seen previously for simpler models, explaining the surprising success of gradient descent in such complicated loss surfaces, but with added structural features that offer explanations for the greater difficulty of training GANs compared to single networks. We have used our model to study the effect of some elementary GAN hyper-parameters and compared with experiments training real GANs on a standard computer vision dataset. We believe that the interesting features of our model, and their correspondence with real GANs, are yet further compelling evidence for the role of statistical physics effects in deep learning and the value of studying such models as proxies for real deep learning models, and in particular the value of concocting more sophisticated models that reflect aspects of modern neural network design and practice.

Our analysis has focused on the annealed complexity of our spin glass model (i.e. taking the logarithm after the expectation) rather than the quenched complexity (i.e. taking the expectation after the logarithm). Ideally one would compute both, as the quenched complexity is often considered to reflect the typical number of stationary points and is bounded above by the annealed complexity. Computing the quenched complexity is typically more challenging than the annealed and such a calculation for our model could be the subject of a further work requiring considerable technical innovations. Even the elegant and very general methods presented recently in [40] are restricted only to the annealed case. Agreement between annealed and quenched is known only in a few special cases closely related to spherical spin glasses [61–63] and is not expected in general [33]. It is conceivable that quenched and annealed complexity agree in the case of our model, as it closely related to spin glasses and possesses no distinguished directions (i.e. spikes) such as are present in [33]. Establishing agreement by existing methods requires analysis of pairs of correlated GOE-like matrices. Such an approach for our model may well require analysis of at least 4 correlated matrices (2 per diagonal block), and quite possibly more, including correlations between blocks. We leave this considerable challenge for future work.

From a mathematical perspective, we have extensively studied the limiting spectral density of a novel random matrix ensemble using supersymmetric methods. In the preparation of this paper, we made considerable efforts to complete the average absolute value determinant calculations directly using a supersymmetric representation, as seen in [13], however this was found to be analytically intractable (as expected), but also extremely troublesome numerically (essentially due to analytically intractable and highly complicated Riemann sheet structure in $\mathbb {C}^2$). We were able to sidestep these issues by instead using a Coulomb gas approximation, whose validity we have rigorously proved using a novel combination of concentration arguments and supersymmetric asymptotic expansions. We have verified with numerical simulations our derived mean spectral density for the relevant Random Matrix Theory ensemble and also the accuracy of the Coulomb gas approximation.

We hope that future work will be inspired to further study models of neural networks such as we have considered here. Practically, it would be exciting to explore the possibility of using our insights into GAN loss surfaces to devise algorithmic methods of avoiding training failure. Mathematically, the local spectral statistics of our random matrix ensemble may be interesting to study, particularly around the cusp where the two disjoint components of the limiting spectral density merge.

## Figures and Tables

**Fig. 10:**
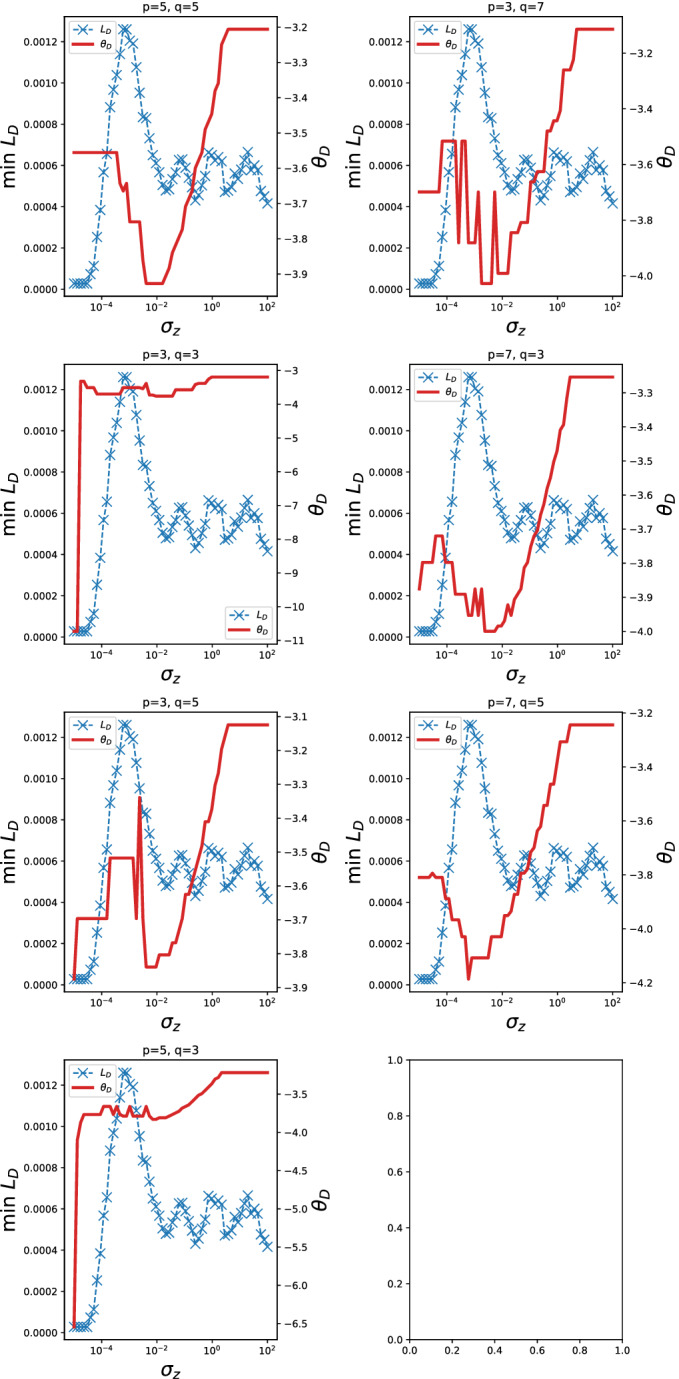
The effect of $\sigma _z$ on minimum $L_D$. Comparison of theoretical predictions of minimum possible discriminator and generator losses to observed minimum losses when training DCGAN on CIFAR10. The blue cross-dashed lines show the experimental DCGAN results, and the solid red show the theoretical results $\vartheta _G, \vartheta _D$. $\kappa =0.5$ is used and *p*, *q* are varied

**Fig. 11:**
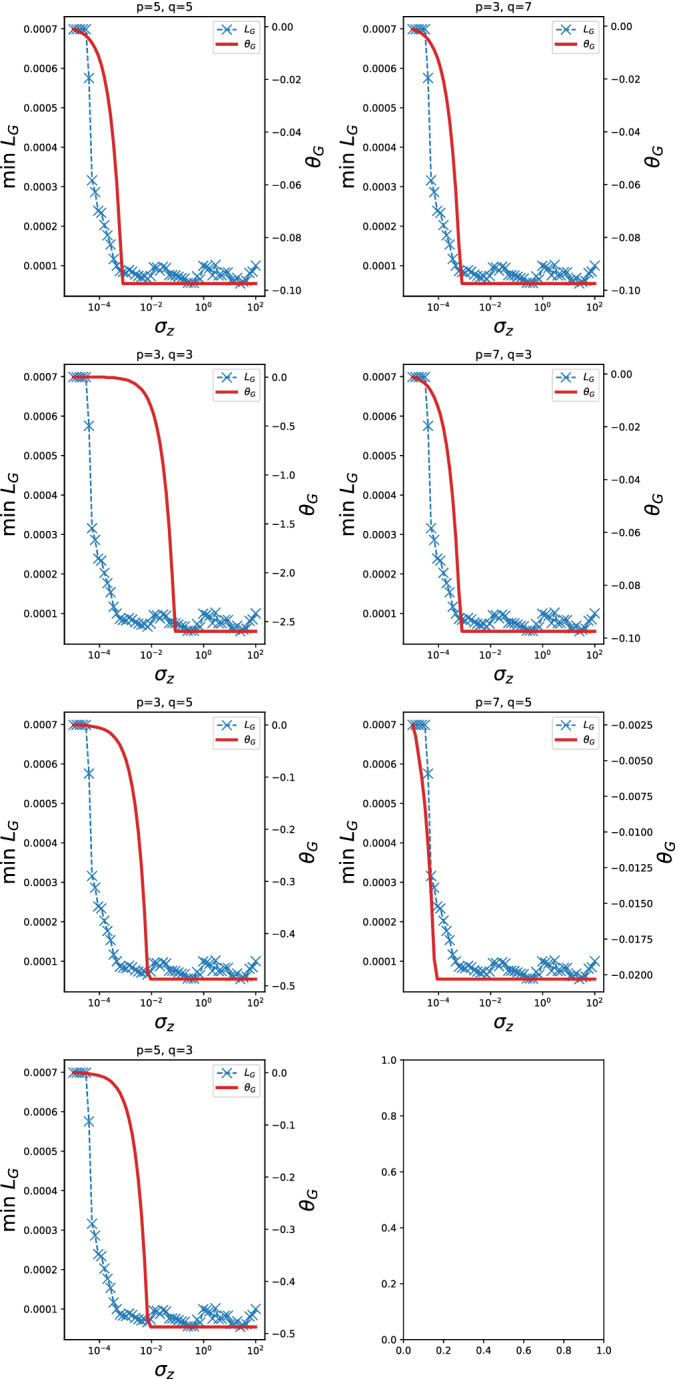
The effect of $\sigma _z$ on minimum $L_G$. Comparison of theoretical predictions of minimum possible discriminator and generator losses to observed minimum losses when training DCGAN on CIFAR10. The blue cross-dashed lines show the experimental DCGAN results, and the solid red show the theoretical results $\vartheta _G, \vartheta _D$. $\kappa =0.5$ is used and *p*, *q* are varied

**Fig. 12:**
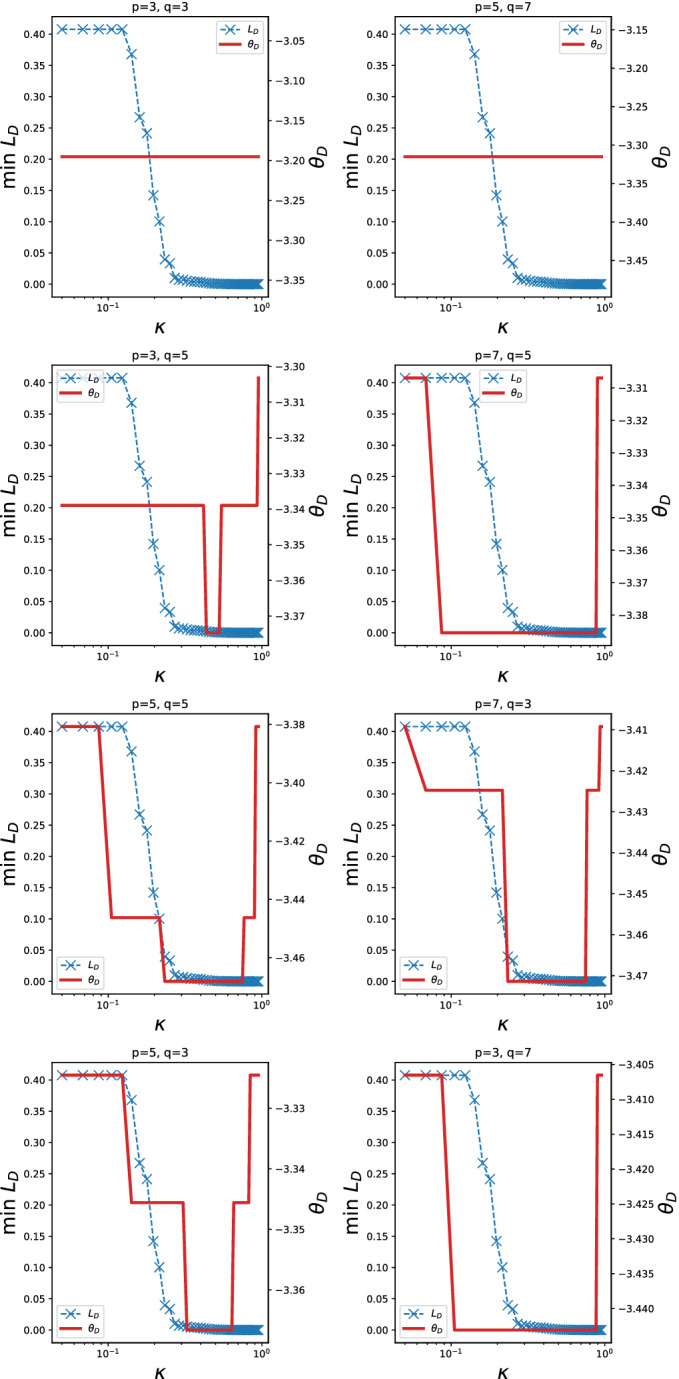
The effect of $\kappa$ on minimum $L_D$. Comparison of theoretical predictions of minimum possible discriminator and generator losses to observed minimum losses when training DCGAN on CIFAR10. The blue cross-dashed lines show the experimental DCGAN results, and the solid red show the theoretical results $\vartheta _G, \vartheta _D$. $\sigma _z=1$ is used and *p*, *q* are varied

**Fig. 13:**
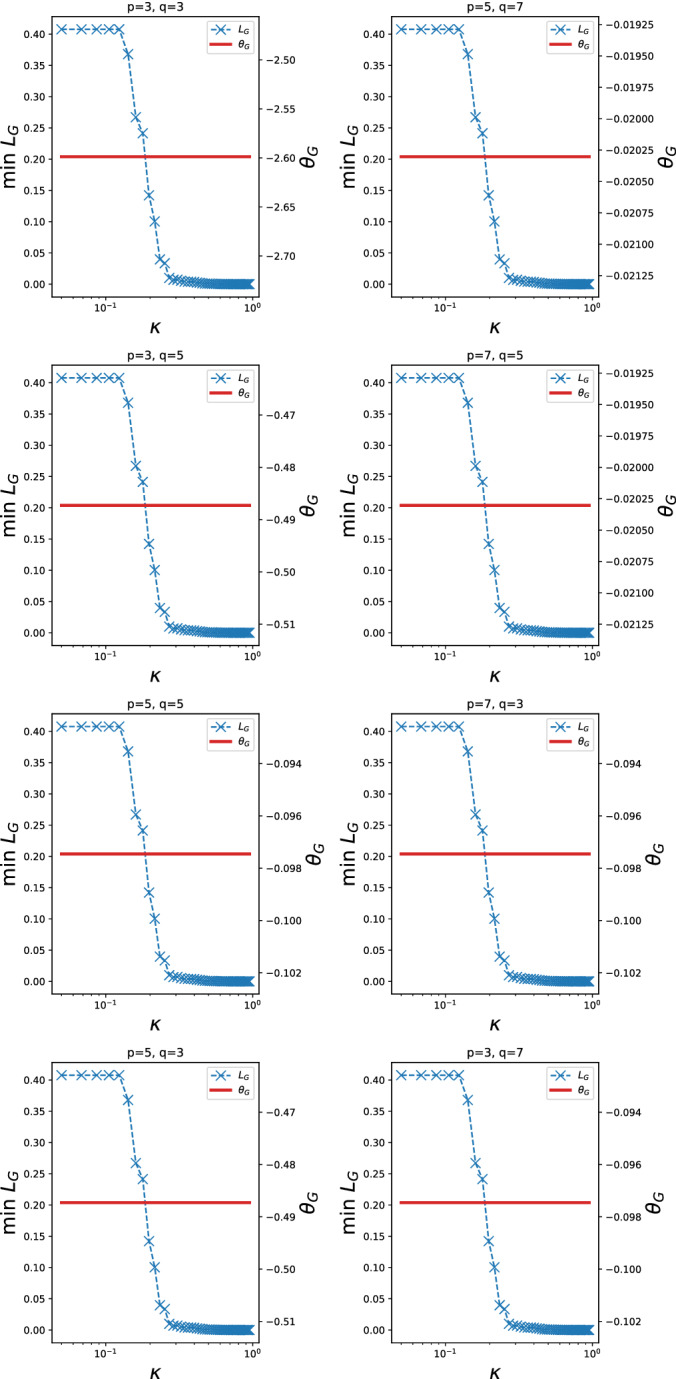
The effect of $\kappa$ on minimum $L_G$. Comparison of theoretical predictions of minimum possible discriminator and generator losses to observed minimum losses when training DCGAN on CIFAR10. The blue cross-dashed lines show the experimental DCGAN results, and the solid red show the theoretical results $\vartheta _G, \vartheta _D$. $\sigma _z=1$ is used and *p*, *q* are varied

## Appendix A: Kac-Rice Results

We repeat here the foundational Kac-Rice result on which our complexity calculations are built.

### Theorem 2

([41] Theorem 12.1.1) Let $\mathcal {M}$ be a compact , oriented, N-dimensional $C^1$ manifold with a $C^1$ Riemannian metric *g*. Let $\phi :\mathcal {M}\rightarrow \mathbb {R}^N$ and $\psi :\mathcal {M}\rightarrow \mathbb {R}^K$ be random fields on $\mathcal {M}$. For an open set $A\subset \mathbb {R}^K$ for which $\partial A$ has dimension $K-1$ and a point ${\varvec{u}}\in \mathbb {R}^{N}$ let

133 $$\begin{aligned} N_{{\varvec{u}}} \equiv \left| \{x\in \mathcal {M} ~|~ \phi (x) = {\varvec{u}}, ~ \psi (x)\in A\}\right| . \end{aligned}$$

Assume that the following conditions are satisfied for some orthonormal frame field E:

- All components of $\phi$, $\nabla _E \phi$, and $\psi$ are a.s. continuous and have finite variances (over $\mathcal {M}$).
- For all $x\in \mathcal {M}$, the marginal densities $p_{x}$ of $\phi (x)$ (implicitly assumed to exist) are continuous at ${\varvec{u}}$.
- The conditional densities $p_{x}(\cdot |\nabla _E\phi (x),\psi (x))$ of $\phi (x)$ given $\psi (x)$ and $\nabla _E\phi (x)$ (implicitly assumed to exist) are bounded above and continuous at ${\varvec{u}}$, uniformly in $\mathcal {M}$.
- The conditional densities $p_x (\cdot |\phi (x) = {\varvec{z}})$ of $\det (\nabla _{E_j}\phi ^i (x))$ given are continuous in a neighbourhood of 0 for ${\varvec{z}}$ in a neighbourhood of ${\varvec{u}}$ uniformly in $\mathcal {M}$.
- The conditional densities $p_x (\cdot |\phi (x) = {\varvec{z}})$ are continuous for ${\varvec{z}}$ in a neighbourhood of ${\varvec{u}}$ uniformly in $\mathcal {M}$.
- The following moment condition holds
  134 $$\begin{aligned} \sup _{x\in \mathcal {M}}\max _{1\le i,j\le N}\mathbb {E}\left\{ \left| \nabla _{E_j}\phi ^i(x)\right| ^N\right\} < \infty \end{aligned}$$
- The moduli of continuity with respect to the (canonical) metric induced by *g* of each component of $\psi$, each component of $\phi$ and each $\nabla _{E_j}\phi ^i$ all satisfy, for any $\epsilon > 0$
  135 $$\begin{aligned} \mathbb {P}( \omega (\eta ) >\epsilon ) = o(\eta ^N), ~~ \text {as } \eta \downarrow 0 \end{aligned}$$
  where the *modulus of continuity* of a real-valued function *G* on a metric space $(T, \tau )$ is defined as (c.f. [41] around (1.3.6))
  136 $$\begin{aligned} \omega (\eta ) :=\sup _{s,t : \tau (s,t)\le \eta }\left| G(s) - G(t)\right| \end{aligned}$$

Then

137 $$\begin{aligned} \mathbb {E}N_{{\varvec{u}}} = \int _{\mathcal {M}}\mathbb {E}\left\{ |\det \nabla _E\phi (x)|\mathbbm {1}\{\psi (x)\in A\} ~| ~ \phi (x) = {\varvec{u}}\right\} p_x({\varvec{u}}) \text {Vol}_g(x) \end{aligned}$$

where $p_x$ is the density of $\phi$ and $\text {Vol}_g$ is the volume element induced by *g* on $\mathcal {M}$.

### Proof of Lemma 1

In the notation of Theorem 2, we make the following choices:

$$\begin{aligned} \phi = \left( \begin{array}{c} \nabla _D L^{(D)}\\ \nabla _G L^{(G)}\end{array}\right) , ~~~ \psi = \left( \begin{array}{c} L^{(D)}\\ L^{(G)}\end{array}\right) \end{aligned}$$

and so

$$\begin{aligned} A = B_D\times B_G, ~~~ {\varvec{u}} = 0. \end{aligned}$$

and the manifold $\mathcal {M}$ is taken to be $S^{N_D}\times S^{N_G}$ with the product topology. It is sufficient to check the conditions of Theorem 2 with the above choices.

Conditions (a)–(f) are satisfied due to Gaussianity and the manifestly smooth definition of $L^{(D)}, L^{(G)}$. The moduli of continuity conditions as in (g) are satisfied separately for $L^{(D)}$ and its derivatives on $S^{N_D}$ and for $L^{(G)}$ and its derivatives on $S^{N_G}$, as seen in the proof of the analogous result for a single spin glass in [2]. But since $\mathcal {M}$ is just a direct product with product topology, it immediately follows that (g) is satisfied, so Theorem 2 applies and we obtain (8). (9) follows simply, using the rules of conditional expectation.

## Appendix B: Gaussian Hessian Calculations

In this section we give the full details of the Gaussian calculations for the distribution of the Hessian:

138 $$\begin{aligned} \left( \begin{array}{cc} \nabla _D^2 L^{(D)}&{} \nabla _{GD} L^{(D)}\\ \nabla _{DG}L^{(G)}&{} \nabla ^2_{G} L^{(G)}\end{array}\right) ~\Bigg |~ \nabla _GL^{(G)}=0, \nabla _DL^{(D)}= 0, L^{(D)}\in B_D, L^{(G)}\in B_G. \end{aligned}$$

These calculations are routine and consist of repeated application of standard results for conditioning multivariate Gaussians, but the details are nevertheless intricate.

Recall the definitions

$$\begin{aligned} L^{(D)}({\varvec{w}}^{(D)}, {\varvec{w}}^{(G)})&= \ell ^{(D)}({\varvec{w}}^{(D)}) - \sigma _z\ell ^{(G)}({\varvec{w}}^{(D)}, {\varvec{w}}^{(G)})\\ L^{(G)}({\varvec{w}}^{(D)}, {\varvec{w}}^{(G)})&= \sigma _z \ell ^{(G)}({\varvec{w}}^{(D)},{\varvec{w}}^{(G)}) \end{aligned}$$

and

$$\begin{aligned} \ell ^{(D)}({\varvec{w}}^{(D)})&= \sum _{i_1,\ldots , i_p=1}^{N_D} X_{i_1,\ldots , i_p} \prod _{k=1}^p w^{(D)}_{i_k}\\ \ell ^{(G)}({\varvec{w}}^{(D)}, {\varvec{w}}^{(G)})&= \sum _{i_1,\ldots , i_{p+q}=1}^{N_D + N_G} Z_{i_1,\ldots , i_{p+q}} \prod _{k=1}^{p+q} w_{i_k} \end{aligned}$$

for i.i.d. Gaussian *X* and *Z*, where ${\varvec{w}}^T = ({{\varvec{w}}^{(D)}}^T, {{\varvec{w}}^{(G)}}^T)$. As mentioned in the main text, we have spherical symmetry in both ${\varvec{w}}^{(D)}$ and ${\varvec{w}}^{(G)}$, so it sufficient to consider the distribution (138) around some fixed specific points on the spheres $S^{N_D}$ and $S^{N_G}$. Following [2], we choose the north poles. We can select a coordinate basis around both poles, e.g. with

$$\begin{aligned} {\varvec{w}}^{(D)}= \left( \sqrt{1 - {\varvec{u}}^2}, {\varvec{u}}\right) , ~~~{\varvec{w}}^{(G)}= \left( \sqrt{1 - {\varvec{v}}^2}, {\varvec{v}}\right) , \end{aligned}$$

for $${\varvec{u}}\in \mathbb {R}^{N_D-1}, {\varvec{v}}\in \mathbb {R}^{N_G - 1}$$ with $${\varvec{u}}^2 \le 1, {\varvec{v}}^2 \le 1$$.

We need the joint distributions

$$\begin{aligned} \left( \ell ^{(D)}, \partial ^{(D)}_i \ell ^{(D)}, \partial ^{(D)}_{jk}\ell ^{(D)}\right) , ~~ \left( \ell ^{(G)}, \partial ^{(G)}_i \ell ^{(G)}, \partial ^{(G)}_{jk}\ell ^{(G)}, \partial ^{(D)}_l \ell ^{(G)}, \partial ^{(D)}_{mn}\ell ^{(G)}\right) \end{aligned}$$

where the two groups are independent from of each other. *The derivatives* $\partial ^{(D)}, \partial ^{(G)}$ *are now Euclidean derivatives with respect to the coordinates* ${\varvec{u}}, {\varvec{v}}$. $\ell ^{(D)}$ behaves just like a single spin glass, and so we have [2]:

139 $$\begin{aligned} Var(\ell ^{(D)})&= 1, \end{aligned}$$

140 $$\begin{aligned} Cov(\partial ^{(D)}_i \ell ^{(D)}, \partial ^{(D)}_{jk} \ell ^{(D)})&= 0, \end{aligned}$$

141 $$\begin{aligned} \partial ^{(D)}_{ij}\ell ^{(D)}~|~ \{\ell ^{(D)}=x_D\}&\sim \sqrt{(N_D-1)p(p-1)}GOE^{N_D - 1} - x_DpI. \end{aligned}$$

To find the joint and thence conditional distributions for $\ell ^{(G)}$, we first note that $\ell ^{(G)}$ is simply a spin glass on a partitioned vector ${\varvec{w}}^T = ({{\varvec{w}}^{(D)}}^T, {{\varvec{w}}^{(G)}}^T)$, so

142 $$\begin{aligned} Cov(\ell ^{(G)}({\varvec{w}}^{(D)}, {\varvec{w}}^{(G)}), \ell ^{(G)}({{\varvec{w}}^{(D)}}', {{\varvec{w}}^{(G)}}')) = \left( {\varvec{w}}^{(D)}\cdot {{\varvec{w}}^{(D)}}' + {\varvec{w}}^{(G)}\cdot {{\varvec{w}}^{(G)}}'\right) ^{p+q} \end{aligned}$$

from which, by comparing with [2], one can obtain the necessary expressions, at the north poles in a coordinate basis. Practically, one writes ${{\varvec{w}}^{(D)}}^T = (\sqrt{1 - \sum _{j}u_j^2}, u_1, \ldots , u_{N_D -1})$, and similarly for ${\varvec{w}}^{(G)}$. Then one takes derivatives of (142) with respect to these new variables around the north poles. Finally, one sets ${\varvec{w}}^{(D)}={{\varvec{w}}^{(D)}}'$ and takes $u_j=0 ~ \forall j$, and similarly for ${\varvec{w}}^{(G)}$. The resulting expressions are largely familiar from the standard spin glass in [2], except there are extra cross terms between ${\varvec{w}}^{(D)}$ and ${\varvec{w}}^{(G)}$:

143 $$\begin{aligned} Var(\ell ^{(G)})&= 2^{p+q},\end{aligned}$$

144 $$\begin{aligned} Cov\left( \partial ^{(G)}_{ij}\ell ^{(G)}, \ell ^{(G)}\right)&= - (p+q)2^{p+q}\delta _{ij},\end{aligned}$$

145 $$\begin{aligned} Cov\left( \partial ^{(D)}_{ij} \ell ^{(G)}, \ell ^{(G)}\right)&= - (p+q)2^{p+q}\delta _{ij},\end{aligned}$$

146 $$\begin{aligned} Cov\left( \partial ^{(G)}_{ij}\ell ^{(G)}, \partial ^{(G)}_{kl}\ell ^{(G)}\right)&= 2^{p+q}\left[ (p+q)(p+q-1)\left( \delta _{ik}\delta _{jl} + \delta _{il}\delta _{jk}\right) \right. \nonumber \\&\quad \left. + (p+q)^2 \delta _{ij}\delta _{kl}\right] ,\end{aligned}$$

147 $$\begin{aligned} Cov\left( \partial ^{(G)}_{ij}\ell ^{(G)}, \partial ^{(D)}_{kl}\ell ^{(G)}\right)&= 2^{p+q} (p+q)^2 \delta _{ij}\delta _{kl},\end{aligned}$$

148 $$\begin{aligned} Cov\left( \partial ^{(G)}_i\partial ^{(D)}_j\ell ^{(G)}, \partial ^{(G)}_{k}\partial ^{(D)}_l\ell ^{(G)}\right)&= 2^{p+q} (p+q)(p+q-1) \delta _{ik}\delta _{jl},\end{aligned}$$

149 $$\begin{aligned} Cov\left( \partial ^{(G)}_{ij}\ell ^{(G)}, \partial ^{(G)}_{k}\partial ^{(D)}_l\ell ^{(G)}\right)&= 0 \end{aligned}$$

150 $$\begin{aligned} Cov\left( \partial ^{(D)}_{ij}\ell ^{(G)}, \partial ^{(D)}_{k}\partial ^{(G)}_l\ell ^{(G)}\right)&= 0,\end{aligned}$$

151 $$\begin{aligned} Cov\left( \partial ^{(D)}_{i}\partial ^{(G)}_j \ell ^{(G)}, \ell ^{(G)}\right)&= 0. \end{aligned}$$

Also, all first derivatives of $\ell ^{(G)}$ are clearly independent of $\ell ^{(G)}$ and its second derivatives by the same reasoning as in [2]. Note that

152 $$\begin{aligned} Cov\left( \partial ^{(D)}_i L^{(D)}, \partial ^{(D)}_j L^{(D)}\right)&= \left( p + \sigma _z^2 2^{p+q}(p+q)\right) \delta _{ij} \end{aligned}$$

153 $$\begin{aligned} Cov\left( \partial ^{(G)}_iL^{(G)}, \partial ^{(G)}_j L^{(G)}\right)&= \sigma ^2_z 2^{p+q}(p+q)\delta _{ij} \end{aligned}$$

154 $$\begin{aligned} Cov\left( \partial ^{(D)}_iL^{(D)}, \partial ^{(G)}_j L^{(G)}\right)&= 0 \end{aligned}$$

and so

155 $$\begin{aligned} \varphi _{\left( \nabla _D L^{(D)}, \nabla _G L^{(G)}\right) }(0) = (2\pi )^{-\frac{N-2}{2}} \left( p + \sigma _z^22^{p+1}(p+q)\right) ^{-\frac{N_D - 1}{2}} \left( \sigma _z^2 2^{p+q} (p+q)\right) ^{-\frac{N_G-1}{2}}. \end{aligned}$$

We need now to calculate the joint distribution of $(\partial ^{(D)}_{ij}\ell ^{(G)}, \partial ^{(G)}_{kl}\ell ^{(G)})$ conditional on $\{\ell ^{(G)}= x_G\}$. Denote the covariance matrix for $(\partial ^{(D)}_{ij}\ell ^{(G)}, \partial ^{(G)}_{kl}\ell ^{(G)}, \ell ^{(G)})$ by

156 $$\begin{aligned} \varSigma = \left( \begin{array}{cc} \varSigma _{11}&{}\varSigma _{12} \\ \varSigma _{21}&{}\varSigma _{22} \end{array}\right) \end{aligned}$$

where

157 $$\begin{aligned} \varSigma _{11}&= 2^{p+q}\left( \begin{array}{cc} (p+1)(p+q-1)(1 + \delta _{ij}) + (p+q)^2\delta _{ij} &{} (p+q)^2 \delta _{ij}\delta _{kl} \\ (p+q)^2 \delta _{ij}\delta _{kl} &{} (p+1)(p+q-1)(1 + \delta _{kl}) + (p+q)^2\delta _{kl} \end{array}\right) , \end{aligned}$$

158 $$\begin{aligned} \varSigma _{12}&= -2^{p+q} (p+q)\left( \begin{array}{c} \delta _{ij} \\ \delta _{kl} \end{array}\right) , \end{aligned}$$

159 $$\begin{aligned} \varSigma _{21}&= -2^{p+q} (p+q)\left( \begin{array}{cc} \delta _{ij}&\delta _{kl} \end{array}\right) , \end{aligned}$$

160 $$\begin{aligned} \varSigma _{22}&= 2^{p+q}. \end{aligned}$$

The conditional covariance is then

161 $$\begin{aligned} \bar{\varSigma } = \varSigma _{11} - \varSigma _{12} \varSigma _{22}^{-1}\varSigma _{21} = 2^{p+q}(p+1)(p+q-1)\left( \begin{array}{cc} 1 + \delta _{ij} &{} 0 \\ 0 &{} 1 + \delta _{kl} \end{array}\right) . \end{aligned}$$

Identical reasoning applied to $(\partial ^{(G)}_{ij}\ell ^{(G)}, \partial ^{(G)}_{kl}\ell ^{(G)}, \ell ^{(G)})$ and $(\partial ^{(D)}_{ij}\ell ^{(G)}, \partial ^{(D)}_{kl}\ell ^{(G)}, \ell ^{(G)})$ shows that, conditional on $\{\ell ^{(G)}= x_G\}$, $\nabla _G^2\ell ^{(G)}$ and $\nabla _D^2\ell ^{(G)}$ have independent entries up-to symmetry, so 161 demonstrates they are independent GOEs and we have:

162 $$\begin{aligned}&\left( \begin{array}{cc} -\nabla _D^2\ell ^{(G)}&{} -\nabla _G\nabla _D \ell ^{(G)}\\ \nabla _D\nabla _G \ell ^{(G)}&{} \nabla ^2 \ell ^{(G)}\end{array}\right) ~|~ \{\ell ^{(G)}= x_G\}\nonumber \\&\quad \overset{d}{=} \sqrt{2^{p+q+1}(p+q)(p+q-1)} \left( \begin{array}{cc} \sqrt{N_D -1}M^{(D)}_1 &{} -2^{-1/2}G \\ 2^{-1/2}G^T &{} \sqrt{N_G - 1}M^{(G)} \end{array}\right) \nonumber \\&\qquad - (p+q)x_G2^{p+1} \left( \begin{array}{cc} -I_{N_D} &{} 0 \\ 0 &{} I_{N_G} \end{array}\right) \end{aligned}$$

where $M^{(D)}_1\sim GOE^{N_D - 1}$ and $M^{(G)} \sim GOE^{N_G - 1}$ are independent GOEs and *G* is an independent $N_D - 1 \times N_G - 1$ Ginibre matrix with entries of unit variance.

## Appendix C: Bipartite Spin-Glass Formulation

Recalling the expression for $\ell ^{(G)}$, one could argue that a more natural formulation would be

$$\begin{aligned} \ell ^{(G)}({\varvec{w}}^{(D)}, {\varvec{w}}^{(G)})&= \sum _{i_1,\ldots , i_p=1}^{N_D} \sum _{j_1,\ldots , j_q=1}^{N_G} Z_{i_1,\ldots , i_p, j_1,\ldots , j_q} \prod _{k=1}^p w^{(D)}_{i_k} \prod _{l=1}^q w^{(G)}_{j_l} \end{aligned}$$

for i.i.d. Gaussian *Z*. In this case, each term in the sum contains exactly *p* weights from the discriminator network and *q* weights from the generator. This object is known as a bipartite spin glass. We will now present the Gaussian calculations. We need the joint distributions

$$\begin{aligned} \left( \ell ^{(D)}, \partial ^{(D)}_i \ell ^{(D)}, \partial ^{(D)}_{jk}\ell ^{(D)}\right) , ~~ \left( \ell ^{(G)}, \partial ^{(G)}_i \ell ^{(G)}, \partial ^{(G)}_{jk}\ell ^{(G)}, \partial ^{(D)}_l \ell ^{(G)}, \partial ^{(D)}_{mn}\ell ^{(G)}\right) \end{aligned}$$

where the two groups are independent from of each other. As in [2], we will simplify the calculation by evaluating in the region of the north poles on each hyper-sphere. $\ell ^{(D)}$ behaves just like a single spin glass, and so we have [2]:

163 $$\begin{aligned} Var(\ell ^{(D)})&= 1,\end{aligned}$$

164 $$\begin{aligned} Cov(\partial ^{(D)}_i \ell ^{(D)}, \partial ^{(D)}_{jk} \ell ^{(D)})&= 0,\end{aligned}$$

165 $$\begin{aligned} \partial ^{(D)}_{ij}\ell ^{(D)}~|~ \{\ell ^{(D)}=x_D\}&\sim \sqrt{(N_D-1)p(p-1)}GOE^{N_D - 1} - x_DpI,\end{aligned}$$

166 $$\begin{aligned} Cov(\partial ^{(D)}_i\ell ^{(D)}, \partial ^{(D)}_j \ell ^{(D)})&= p\delta _{ij}. \end{aligned}$$

To find the joint and thence conditional distributions for $\ell ^{(G)}$, we first compute the covariance function, which follows from the independence of the *Z*:

167 $$\begin{aligned}&Cov(\ell ^{(G)}({\varvec{w}}^{(D)}, {\varvec{w}}^{(G)}), \ell ^{(G)}({{\varvec{w}}^{(D)}}', {{\varvec{w}}^{(G)}}'))\end{aligned}$$

168 $$\begin{aligned}&=\sum _{\begin{array}{c} i_1,\ldots , i_p=1\\ i_1',\ldots , i_p'=1 \end{array}}^{N_D} ~~\sum _{\begin{array}{c} j_1,\ldots , j_q=1\\ j_1',\ldots , j_q'=1 \end{array}}^{N_G} \mathbb {E} Z_{{\varvec{i}}{\varvec{i}}}Z_{{\varvec{i}}'{\varvec{j}}'} \prod _{k=1}^p w^{(D)}_{i_k}{w^{(D)}_{i_k'}}' \prod _{l=1}^q w^{(G)}_{j_l}{w^{(G)}_{j_l'}}'\end{aligned}$$

169 $$\begin{aligned}&=\sum _{i_1,\ldots , i_p=1}^{N_D} ~~\sum _{j_1,\ldots , j_q=1}^{N_G} \prod _{k=1}^p w^{(D)}_{i_k}{w^{(D)}_{i_k}}' \prod _{l=1}^q w^{(G)}_{j_l}{w^{(G)}_{j_l}}'\end{aligned}$$

170 $$\begin{aligned}&= \left( {\varvec{w}}^{(D)}\cdot {{\varvec{w}}^{(D)}}'\right) ^p({\varvec{w}}^{(G)}\cdot {{\varvec{w}}^{(G)}}')^q \end{aligned}$$

The product structure of the covariance function implies that we can write down the following covariances directly from the simple spin-glass case, as the $\partial ^{(D)}$ and $\partial ^{(G)}$ derivatives act independently on their respective terms:

171 $$\begin{aligned} Var(\ell ^{(G)})&= 1, \end{aligned}$$

172 $$\begin{aligned} Cov\left( \partial ^{(G)}_{ij}\ell ^{(G)}, \ell ^{(G)}\right)&= -q\delta _{ij},\end{aligned}$$

173 $$\begin{aligned} Cov\left( \partial ^{(D)}_{ij} \ell ^{(G)}, \ell ^{(G)}\right)&= -p\delta _{ij},\end{aligned}$$

174 $$\begin{aligned} Cov\left( \partial ^{(G)}_{ij}\ell ^{(G)}, \partial ^{(G)}_{kl}\ell ^{(G)}\right)&= q(q-1)\left( \delta _{ik}\delta _{jl} + \delta _{il}\delta _{jk}\right) + q^2 \delta _{ij}\delta _{kl},\end{aligned}$$

175 $$\begin{aligned} Cov\left( \partial ^{(D)}_{ij}\ell ^{(G)}, \partial ^{(D)}_{kl}\ell ^{(G)}\right)&= p(p-1)\left( \delta _{ik}\delta _{jl} + \delta _{il}\delta _{jk}\right) + p^2 \delta _{ij}\delta _{kl},\end{aligned}$$

176 $$\begin{aligned} Cov\left( \partial ^{(G)}_{ij}\ell ^{(G)}, \partial ^{(D)}_{kl}\ell ^{(G)}\right)&= pq \delta _{ij}\delta _{kl},\end{aligned}$$

177 $$\begin{aligned} Cov\left( \partial ^{(G)}_i\partial ^{(D)}_j\ell ^{(G)}, \partial ^{(G)}_{k}\partial ^{(D)}_l\ell ^{(G)}\right)&= pq \delta _{ik}\delta _{jl},\end{aligned}$$

178 $$\begin{aligned} Cov\left( \partial ^{(G)}_{ij}\ell ^{(G)}, \partial ^{(G)}_{k}\partial ^{(D)}_l\ell ^{(G)}\right)&= 0\end{aligned}$$

179 $$\begin{aligned} Cov\left( \partial ^{(D)}_{ij}\ell ^{(G)}, \partial ^{(D)}_{k}\partial ^{(G)}_l\ell ^{(G)}\right)&= 0,\end{aligned}$$

180 $$\begin{aligned} Cov\left( \partial ^{(D)}_{i}\partial ^{(G)}_j \ell ^{(G)}, \ell ^{(G)}\right)&= 0. \end{aligned}$$

Also, all first derivatives of $\ell ^{(G)}$ are clearly independent of $\ell ^{(G)}$ and its second derivatives by the same reasoning and

181 $$\begin{aligned} Cov\left( \partial ^{(G)}_{i}\ell ^{(G)}, \partial ^{(G)}_j\ell ^{(G)}\right)&= q\delta _{ij},\end{aligned}$$

182 $$\begin{aligned} Cov\left( \partial ^{(D)}_{i}\ell ^{(G)}, \partial ^{(D)}_j\ell ^{(G)}\right)&= p\delta _{ij},\end{aligned}$$

183 $$\begin{aligned} Cov\left( \partial ^{(D)}_{i}\ell ^{(G)}, \partial ^{(G)}_j\ell ^{(G)}\right)&= 0. \end{aligned}$$

We caw deduce the full gradient covariances, recalling that $\ell ^{(D)}$ and $\ell ^{(G)}$ are independent:

184 $$\begin{aligned} Cov\left( \partial ^{(D)}_i L^{(D)}, \partial ^{(D)}_j L^{(D)}\right)&= p\left( 1 + \sigma _z^2\right) \delta _{ij}\end{aligned}$$

185 $$\begin{aligned} Cov\left( \partial ^{(G)}_iL^{(G)}, \partial ^{(G)}_j L^{(G)}\right)&= \sigma ^2_z q\delta _{ij}\end{aligned}$$

186 $$\begin{aligned} Cov\left( \partial ^{(D)}_iL^{(D)}, \partial ^{(G)}_j L^{(G)}\right)&= 0 \end{aligned}$$

and so

187 $$\begin{aligned} \varphi _{\left( \nabla _D L^{(D)}, \nabla _G L^{(G)}\right) }(0) = (2\pi )^{-\frac{N-2}{2}} \left( p + \sigma _z^2p\right) ^{-\frac{N_D - 1}{2}} \left( \sigma _z^2 q\right) ^{-\frac{N_G-1}{2}}. \end{aligned}$$

We need now to calculate the joint distribution of $(\partial ^{(D)}_{ij}\ell ^{(G)}, \partial ^{(G)}_{kl}\ell ^{(G)})$ conditional on $\{\ell ^{(G)}= x_G\}$. Denote the covariance matrix for $(\partial ^{(D)}_{ij}\ell ^{(G)}, \partial ^{(G)}_{kl}\ell ^{(G)}, \ell ^{(G)})$ by

188 $$\begin{aligned} \varSigma = \left( \begin{array}{cc} \varSigma _{11}&{}\varSigma _{12} \\ \varSigma _{21}&{}\varSigma _{22} \end{array}\right) \end{aligned}$$

where

189 $$\begin{aligned} \varSigma _{11}&= \left( \begin{array}{cc} p(p-1)(1 + \delta _{ij}) + p^2\delta _{ij} &{} pq\delta _{ij}\delta _{kl} \\ pq \delta _{ij}\delta _{kl} &{} q(q-1)(1 + \delta _{kl}) + q^2\delta _{kl} \end{array}\right) ,\end{aligned}$$

190 $$\begin{aligned} \varSigma _{12}&= -\left( \begin{array}{c} p\delta _{ij} \\ q\delta _{kl} \end{array}\right) ,\end{aligned}$$

191 $$\begin{aligned} \varSigma _{21}&= -\left( \begin{array}{cc} p\delta _{ij}&q\delta _{kl} \end{array}\right) ,\end{aligned}$$

192 $$\begin{aligned} \varSigma _{22}&= 1. \end{aligned}$$

The conditional covariance is then

193 $$\begin{aligned} \bar{\varSigma }&= \varSigma _{11} - \varSigma _{12}\varSigma _{22}^{-1}\varSigma _{21}\end{aligned}$$

194 $$\begin{aligned}&= \left( \begin{array}{cc} p(p-1)(1+\delta _{ij}) &{} 0\\ 0 &{} q(q-1) (1 + \delta _{kl}) \end{array}\right) . \end{aligned}$$

Repeating this calculation for $(\partial ^{(G)}_{ij}\ell ^{(G)}, \partial ^{(G)}_{kl}\ell ^{(G)}, \ell ^{(G)})$ demonstrates that $\nabla _G^2\ell ^{(G)}\mid \{\ell ^{(G)}= x_G\}$ has independent entries, up-to symmetry. The result (194) demonstrates that, conditional on $\{\ell ^{(G)}= x_G\}$, $\nabla _G^2\ell ^{(G)}$ and $\nabla _D^2\ell ^{(G)}$ are independent GOEs. In summary, from (194) and (177–179) we obtain

195 $$\begin{aligned}&\left( \begin{array}{cc} -\nabla _D^2\ell ^{(G)}&{} -\nabla _G\nabla _D \ell ^{(G)}\\ \nabla _D\nabla _G \ell ^{(G)}&{} \nabla ^2 \ell ^{(G)}\end{array}\right) ~|~ \{\ell ^{(G)}= x_G\}\nonumber \\&\quad \overset{d}{=} \sqrt{2}\left( \begin{array}{cc} \sqrt{N_D -1}\sqrt{p(p-1)}M^{(D)} &{} -2^{-1/2}\sqrt{pq}G \\ 2^{-1/2}\sqrt{pq}G^T &{} \sqrt{N_G - 1}\sqrt{q(q-1)}M^{(G)} \end{array}\right) \nonumber \\&\qquad - x_G \left( \begin{array}{cc} -pI_{N_D} &{} 0 \\ 0 &{} qI_{N_G} \end{array}\right) \end{aligned}$$

where $M^{(D)}\sim GOE^{N_D - 1}$ and $M^{(G)} \sim GOE^{N_G - 1}$ are independent GOEs and *G* is an independent $N_D - 1 \times N_G - 1$ Ginibre matrix with entries of unit variance.

At this point a problem becomes apparent. Suppose that $q\le p$, then the variance of the lower-right block is strictly less than that of the off diagonal blocks. If we proceed with the strategy in the main text, there is no way of decomposing the lower-right block as a sum of two independent smaller variance GOEs with one matching the variance of the off diagonal blocks. Similarly, if $q>p$, then the final Hessian involving $L^{(D)}, L^{(G)}$ will have lower-variance in the upper-left block than the off-diagonals unless very specific undesirable conditions hold on *p*, *q* and $\sigma _z$. In either of these cases, we cannot decompose the final Hessian as a sum of a large $N-2\times N-2$ GOE and some smaller GOEs in the upper-left or lower-right blocks. We would therefore have to truly compute the Ginibre averages in the supersymmetric method, which we believe is intractable.

We could complete the complexity calculation via the methods of this paper supposing that the appropriate conditions hold on *p*, *q* and $\sigma _z$. It would look much the same as the calculation in the main text, though the resulting polynomial for the spectral density would be different. Since this work was completed, the complexity results for bipartite spin glasses were obtained in [64] using an entirely new method developed in the companion paper [40]. Applying this method arguably presents more technical hurdles than the supersymmetric approach to complexity calculations, however it is much more general and can be applied to the above model for any *p*, *q* and $\sigma _z$.

## Appendix D: Extra Plots

This section contains some extra plots to back up the comparisons between our model’s predictions and the experimental DCGAN results in Sect. 6.2 (Figs. 10, 11, 12, 13). In particular, we produce versions of the plots in Figs. 8 and 9 but for various values of *p* and *q* other than $p=q=5$. Since $p=q=5$ is the structurally correct choice for the DCGAN, it is natural to ask if any agreement between theory and experiment is most closely obtained with $p=q=5$. Figure 13 shows that the model has the same deficiency in $\kappa$ for all *p*, *q* values tested. Figure 12 shows best agreement for $p=q=5$, $p=3, q=7$ and $p=7, q=3$, and similarly in Fig. 11. There is perhaps weak evidence that the role of *p* and *q* as representing the number of layers in the networks has some merit experimentally.

## Footnotes

1 https://github.com/npbaskerville/loss-surfaces-of-gans.

2 We use the convention of the *M*-sphere being the sphere embedded in $\mathbb {R}^M$.

3 Number of filters in a layer is either proportional to $n_D$ or $n_D^2$ depending on the layer (and similarly with $n_G$).
